# Photoswitchable upconversion nanoparticles with excitation-dependent emission for programmed stepwise NIR phototherapy

**DOI:** 10.1016/j.isci.2023.107859

**Published:** 2023-09-09

**Authors:** Shanshan Zheng, Hengji Zhang, Ting Sheng, Yi Xiang, Jing Wang, Yao Tang, Yihan Wu, Jinliang Liu, Xiaohui Zhu, Yong Zhang

**Affiliations:** 1 School of Environmental and Chemical Engineering, Shanghai University, Shanghai 200444, China; 2 China Steel Development Research Institute, Beijing 100029, China; 3 Department of Biomedical Engineering, College of Design and Engineering, National University of Singapore, Singapore 117583, Singapore

**Keywords:** Therapeutics, Drug delivery system, Nanoparticles, Cancer

## Abstract

Programmable control over therapeutic processes in phototherapy, like photodynamic therapy (PDT), is promising but challenging. This study uses an energy segmentation-based strategy to synthesize core-multi-shell upconversion nanoparticles (UCNPs), which can release three different colors (red, green, and blue) upon exposure to different near-infrared light (1550 nm, 808 nm, and 980 nm). By combining these UCNPs with photosensitizers and nitric oxide (NO) donors, a smart “off-on” PDT nanoplatform is developed. UCNPs enable independent activation of imaging, release of NO, and generation of reactive oxygen species using specific light wavelengths. The results show that sequential NO release before PDT can greatly alleviate tumor hypoxia by reducing oxygen consumption. This stepwise approach shows potential for precise NIR light-activated and imaging-guided phototherapy.

## Highlights

• An energy segmentation-based strategy is proposed to develop photoswitchable UCNPs
• The developed UCNPs enable controlled activation of imaging, gas therapy, and PDT
• Tumor hypoxia issue can be alleviated, showing its strong therapeutic promise

## Graphical abstract

**Figure:**
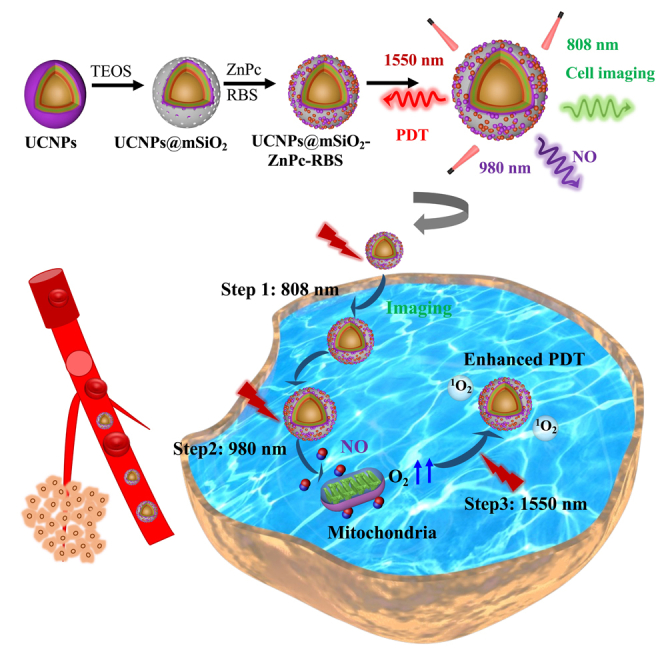

## Introduction

Photodynamic therapy (PDT) is a type of light therapy that is widely used to treat various malignant diseases with minimal invasiveness and toxicity.^1^^,^^2^^,^^3^ Typically, three elements are involved in a PDT process^4^^,^^5^: excitation light, photosensitizing molecules (photosensitizers), and oxygen. Upon irradiation with light of an appropriate wavelength, the photosensitizer may undergo a transition from an excited singlet state to a triplet state, which may then react with nearby oxygen (O_2_) and produce singlet molecular oxygen.^2^^,^^6^ As the main reactive oxygen species (ROS), these highly cytotoxic singlet oxygen molecules can cause irreversible cellular damage and induce cell death or tumor destruction.^7^^,^^8^ Despite its great achievements, PDT still faces several challenges. Firstly, commonly used photosensitizers are mainly activated by ultraviolet or visible light with limited tissue penetration depth, which in turn restricts the application of PDT to superficial lesions.^9^^,^^10^^,^^11^ Secondly, as an oxygen-dependent therapy, the therapeutic efficiency of PDT largely depends on the presence of ambient oxygen.^4^^,^^12^ For example, the yield of singlet oxygen of aloe emodin (AE) photosensitizers may drop to nearly zero when oxygen is absent. Unfortunately, the tumor microenvironment is usually in a hypoxic state due to unbalanced oxygen supply.^13^ Furthermore, damage to the vasculature and continuous depletion of oxygen during PDT may even further exacerbate hypoxia. Therefore, hypoxia is considered as a causative factor for tumor resistance to PDT and many other therapeutic strategies including chemotherapy, radiotherapy, etc.^14^^,^^15^^,^^16^

Lanthanide-ion doped upconversion nanoparticles (UCNPs) can convert long-wavelength excitation light (e.g., NIR) to short-wavelength emission light (UV or visible).^17^^,^^18^^,^^19^^,^^20^ Since NIR light has a deeper tissue penetration depth than UV and visible light due to the minimal absorbance of biomolecules in the NIR spectral window (700–1000 nm),^21^^,^^22^^,^^23^ UCNPs can be used as light transducer to excite photosensitizes in deep tissues. Various photosensitizers, e.g., zinc phthalocyanine (ZnPc),^11^^,^^24^^,^^25^ merocyanine 540 (MC540),^26^^,^^27^ chlorin e6 (Ce6),^28^^,^^29^^,^^30^ rose bengal,^31^^,^^32^^,^^33^ curcumin,^6^ TiO_2_,^34^ and black phosphorus nanosheets (BPNS),^35^ have been combined with UCNPs and demonstrated great potentials for deep-tissue PDT applications. In addition to improving the tissue penetration of light, many efforts have been devoted to overcoming other obstacles in PDT, namely hypoxia and on-demand activation.^11^^,^^24^^,^^36^^,^^37^^,^^38^^,^^39^ For example, MnO_2_ layer^37^ and Fe(OH)_3_^38^ compounds have been used in UCNP-based PDT because these nanocatalysts can regulate hypoxic tumor microenvironment by decomposing intracellular hydrogen peroxide (H_2_O_2_). Erythrocyte-sized hemoglobin microgels^36^ have also been combined with UCNPs because they can generate oxygen, heat and ROS to suppress hypoxic tumor. In other studies, orthogonal emissive UCNPs have been used for imaging-guided PDT treatment,^11^^,^^24^ where one emission is used to trigger the release of ROS while the other is used for diagnostic purpose.

**Scheme 1:**
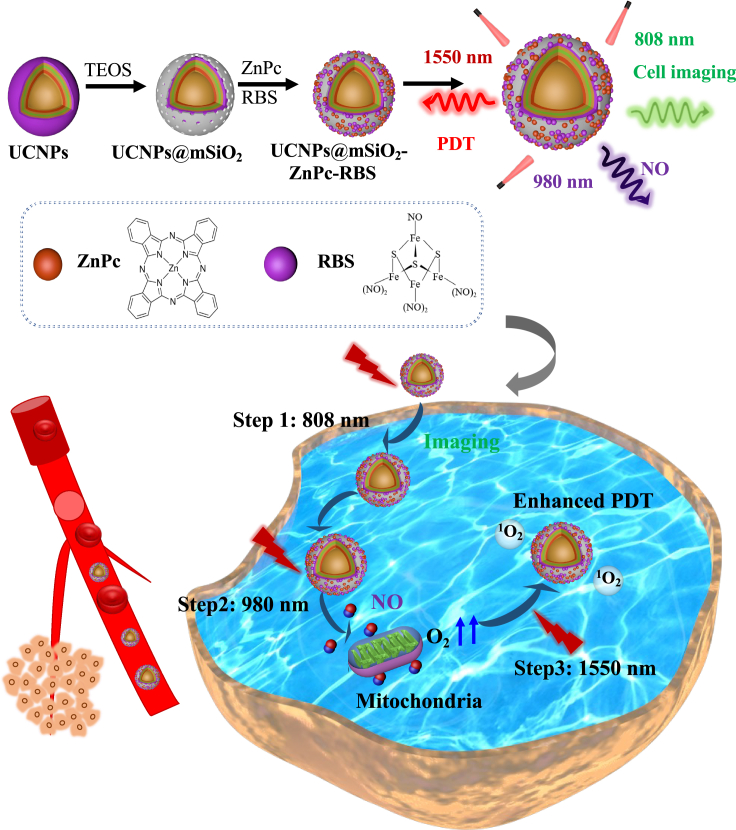
Programmed photodynamic therapy based on photoswitchable upconversion nanoparticles Schematic diagram of cascaded photodynamic therapy via programmable photorelease of nitric oxide (NO) and reactive oxygen species (ROS) enabled by photoswitchable upconversion nanoparticles (UCNPs).

Ideally, the PDT process should be properly controlled and optimized to deliver the photosensitizer to the lesion, subsequently suppress tumor hypoxia, and then release ROS in a step-by-step manner. In other words, imaging guidance, hypoxia suppression and ROS release should be performed or triggered in sequence, giving the right treatment at the right time and for the right duration. However, such programmable PDT requires independent and sequential photoactivation of different therapeutic processes using different excitation lights, which cannot be achieved using conventional UCNPs excitable at a single wavelength only. In this study, an energy segmentation-based strategy is proposed to synthesize core-shell UCNPs (NaYF_4_@NaErF_4_:Tm@NaYF_4_@NaYF_4_:Yb,Ho,Nd@NaYF_4_@NaYF_4_:Yb,Tm) with excitation wavelength-dependent emission, i.e., red emission when excited at 1550 nm, green emission when excited at 808 nm, and blue emission when excited at 980 nm. By carefully tuning the thickness of the luminescent shell and inert shell, the excitation and emission of the three luminescent ions are strictly segmented in different shell regions. Therefore, unwanted crosstalk between these ions can be efficiently avoided and high color purity can be obtained. The specially designed UCNPs are loaded with the photosensitizer zinc phthalocyanine (ZnPc) and nitric oxide (NO) donor Roussin’s black salt (RBS) (Scheme 1). Under 980 nm excitation, the blue emission of UCNPs can excite the RBS salt to produce NO gas. The released NO will inhibit cellular respiration by competing with oxygen for binding to mitochondria so less oxygen is consumed. When the excitation wavelength is switched to 1550 nm, ZnPc is then activated by the red emission of UCNPs. Since tumor oxygenation is greatly increased by inhibiting mitochondrial respiration, the tumor hypoxic environment is relieved and more ROS can be generated. The green emission of UCNPs under 808 nm excitation is used for imaging guidance and identification of lesion areas, as no ROS or NO is generated. It is important to note that the release of NO and ROS must be activated in a cascaded manner. If they are activated simultaneously, there is not enough time to inhibit cellular respiration and PDT will still suffer from hypoxia. Our results demonstrate that precise control of the PDT process and stepwise activation of NO release and ROS production can significantly reduce cancer cell viability and limit tumor growth.

## Results

**Figure 1:**
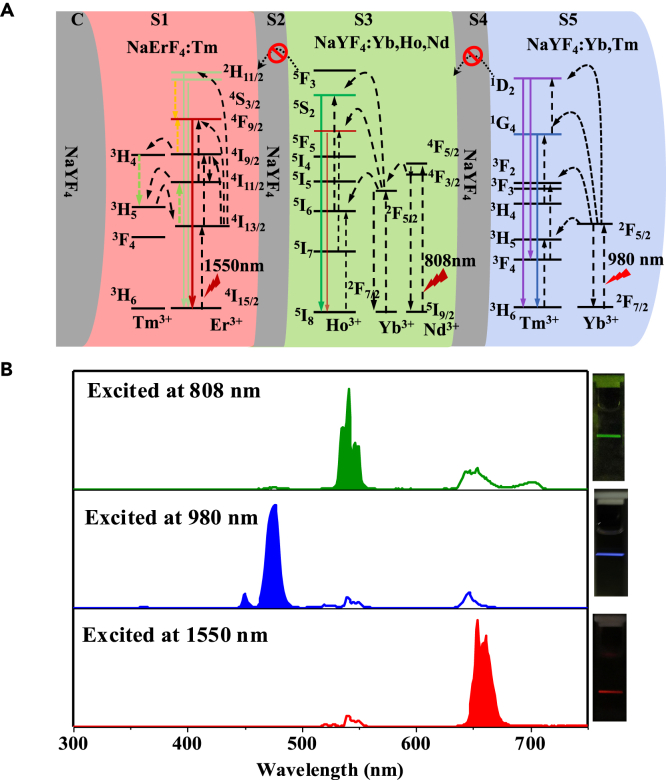
Energy migration pathways and upconversion luminescence spectra of UCNPs under various light excitations (A and B) Schematic illustration of the energy migration pathways (A) and upconversion luminescence spectra (B) of UCNPs under 1550 nm, 980 nm and 808 nm light excitations. The inset in (B) is the luminescence photo of the UCNP solution under illumination with different excitation wavelengths.

The structure of as-developed photoswitchable UCNPs, NaYF_4_@NaErF_4_:Tm@NaYF_4_@NaYF_4_:Yb,Ho,Nd@NaYF_4_@NaYF_4_:Yb,Tm (C@S1@S2@S3@S4@S5), can be divided into three luminescent and three inert regions. As shown in Figure 1A, NaErF_4_:0.5%Tm (S1) layer can be activated under 1550 nm excitation and release red emission, while NaYF_4_:20%Yb,2%Ho,10%Nd (S3) shell emits green signal in response to 808 nm excitation and NaYF_4_:Yb,0.5%Tm (S5) layer releases blue light under 980 nm excitation. Although the NaYF_4_ shell is optically inactive, it is mainly used to enhance emission intensity and block energy transfer processes between different luminescent layers. As for the structure arrangement, since Er^3+^ ions can sensitize 808 nm, 980 nm, and 1550 nm excitation light (Supporting information Figure S1A), the Er^3+^ doped layer is particularly placed in the inner section of the whole core-multi-shell nanostructure. Figure S1B demonstrates a scenario where S1 and S5 shell are replaced with each other, i.e., NaErF_4_ shell in the outermost region and NaYF_4_:Yb, Tm layer in the first shell. In this case, the NaErF_4_ shell in the outermost region would always be the first to be excited, resulting in the presence of red emission regardless of the excitation light used (Figure S1A). This would significantly affect the color purity. However, in Figure S1C, we arranged the shells differently, placing NaErF_4_ in the S1 shell, NaYF_4_:Yb,Nd,Ho in the S3 shell, and NaYF_4_:Yb,Tm in the S5 shell. This is because the green luminescent layer, NaYF_4_:Yb,Ho,Nd (S3), or blue luminescent layer, NaYF_4_:Yb,Tm (S5), can function as a filter that, respectively, prevents 808 nm and 980 nm light from accessing to S1 layer. Under this circumstance, Er^3+^ ions in S1 shell can only respond to 1550 nm light and release red emission with high color purity. Besides, the Tm^3+^ dopant ion can further promote the red color emission of Er^3+^ ions via back-energy transfer between ^3^H_5_ state of Tm^3+^ and ^4^H_13/2_ states of Er^3+^.^40^ As the pair of Nd^3+^-Yb^3+^ ions in the green emitting layer (S3) can respond to both 808 nm and 980 nm light, the S3 shell is then placed in the middle part of the whole nanostructure such that the incident 980 nm light can be blocked by the outermost blue emitting layer (S5). In the S3 shell, Nd^3+^ ions can sensitize 808 nm light and transfer the excitation energy to neighboring Yb^3+^ ions, then to Ho^3+^ activator ions for green emission. Moreover, the thickness of the S3 shell can be varied to regulate the amount of 808 nm photons reaching the S1 shell and control the color output. For the S5 part, the Yb^3+^ sensitizers absorb 980 nm photons and transfer the energy to Tm^3+^ activators for the release of blue upconversion emission. Similar to S3, the S5 layer is also used as the 980 nm photon filter because its thickness can be tuned to confine migration of excitation energy within the S5 shell. By carefully optimizing the thickness of luminescent and inert shell, energy migration pathways upon different excitation lights (1550 nm, 980 nm, and 808 nm) can be precisely segmented and confined in the respective shell, which greatly favors the orthogonal red-green-blue upconversion luminescence (Figure 1B).

**Figure 2:**
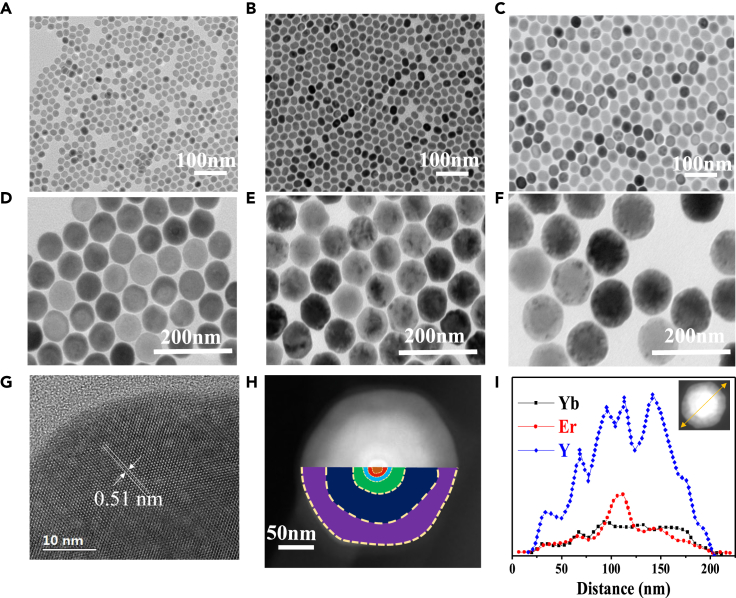
TEM characterization of core-multi-shell UCNP nanoparticle and elemental analysis (A–F) TEM image of Core (A), Core@S1 (B), Core@S1@S2 (C), Core@S1@S2@S3 (D), Core@S1@S2@S3@S4 (E) and Core@S1@S2@S3@S4@S5 (F). (G) High-resolution TEM image of a single UCNP nanoparticle. (H) The HAADF-STEM image of a UCNP nanoparticle. Different colors are used to label core and different shell regions. (I) Energy dispersive x-ray (EDX) line profile analysis of a single UCNP nanoparticle.

Figures 2A–2F present transmission electron microscopy (TEM) images of core and core-multi-shell nanoparticles, which reveal that as-synthesized UCNPs exhibit uniform size and shape. The lattice spacing of the core-quintuple-shell UCNPs is calculated to be 0.51 nm that corresponds to the (100) crystal plane of the hexagonal phase (Figure 2G). Figure S2 presents the size distribution of these particles with different shell thickness. The X-ray diffraction (XRD) also confirms that Core, Core@S1, Core@S1@S2, Core@S1@S2@S3, Core@S1@S2@S3@S4, and Core@S1@S2@S3@S4@S5, nanoparticles all exhibit hexagonal phase (Figure S3). The Scherrer equation, represented as follows:

(Equation 1) $$D=\frac{k\lambda }{\beta \;\cos\;\theta }$$

is commonly used to evaluate the relationship between crystalline domain size (D), broadening of a peak at a particular angle (θ), and the width of the peak at half of its height (β). The particle size increases with continuous coating of different shells. Consequently, it is expected that the half full bandwidth in the XRD peaks is expected to become narrower, which is not observed in Figure S3. There are several reasons for this inconsistence. Firstly, it should be noted that particle size does not necessarily correspond to the crystalline size, as particles may contain multiple crystalline domains. Additionally, the degree of crystallinity is reduced after several layers are deposited on the core, which may cause the broadening of the diffraction peak.

Because the contrast of the HAADF-STEM (high angle annular dark field-scanning transmission electron microscopy) image is sensitive to the atomic number (*Z*) of chemical element, HAADF-STEM technique is further used to identify the morphology of core-quintuple-shell UCNPs. As shown in Figure 2H, bright and dark shells can be observed that corresponds to heavy and light elements present in the nanoparticle, indicating the formation of core-multi-shell nanostructure. This can also be verified by the variation of EDX (energy dispersive x-ray) line profile analysis across the whole nanoparticle (Figure 2I). Notably, the nominal chemical composition of the particle is NaYF_4_@NaErF_4_:Tm@NaYF_4_@NaYF_4_:Yb,Ho,Nd@NaYF_4_@NaYF_4_:Yb,Tm. Therefore, if all the elements in the core or shell region were strictly confined, Er and Yb elements should only be localized in a relatively inner and outer region. However, it is clear that Er and Yb elements are detected almost throughout the whole particle, indicating that the cations are inter-diffused during the synthesis procedure.^41^

**Figure 3:**
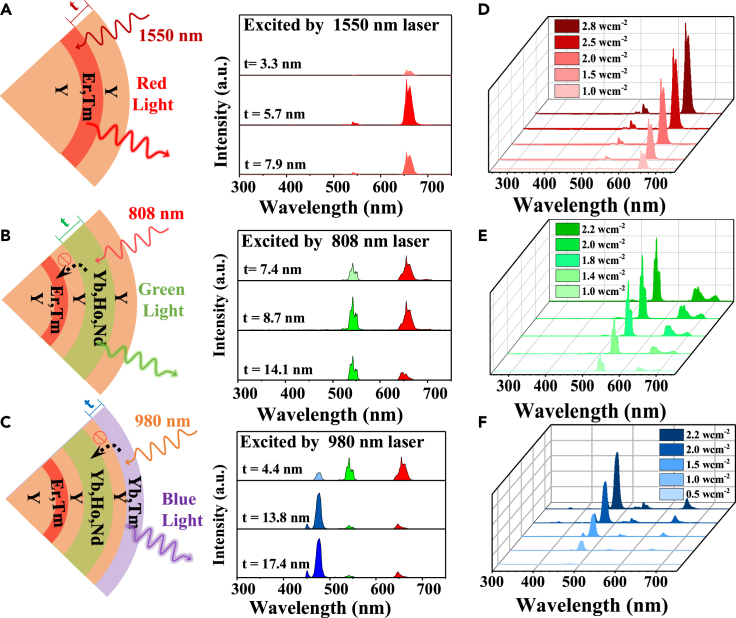
Upconversion spectra of UCNPs with different shell thickness (A) Upconversion luminescence spectra of Core@S1@S2 nanoparticles with different NaYF_4_ (S2) layer thickness under 1550 nm excitation. (B) Upconversion luminescence spectra of Core@S1@S2@S3@S4 nanoparticles with different NaYF_4_:Yb,Ho,Nd (S3) layer thickness under 808 nm excitation. (C) Upconversion luminescence spectra of Core@S1@S2@S3@S4@S5 nanoparticles with different NaYF_4_:Yb,Tm (S5) layer thickness under excitation of 980 nm. (D–F) Upconversion luminescence intensity of UCNPs at different power densities under 1550 nm (D), 808 nm (E), and 980 nm (F) light excitation.

As mentioned previously, the Core@S1@S2(NaYF_4_@NaErF_4_@NaYF_4_) part can emit red luminescence in response to 1550 nm excitation. Specifically, as the thickness of S2 shell is increased from 3.3 nm to 5.7 nm (Figure 3A), the intensity is enhanced by 9.8 times (Figure 3A). This is because the increase of the S2 shell enhances the distance between luminescence center and surrounding quenchers, which reduces the energy transfer between them and accordingly increases the red upconversion luminescence. However, if the thickness of S2 shell is further increased to 7.9 nm, the luminescence intensity is reduced (Figure 3A). This reduction in intensity can be explained by the increased accumulation of internal quenchers from the interfaces between sequentially coated shells, as previously reported.^42^ It is believed that these internal quenchers act as non-radiative quenching channels, leading to a reduction in upconversion intensity when the shell thickness is too thick. On the successful tuning of the red emission, the green-emitting layer, NaYF_4_:Yb,Ho,Nd (S3) is subsequently coated, which can absorb the 808 nm excitation light and generate green upconversion luminescence. Notably, the pre-existing S1 shell (NaErF_4_:Tm) can also sensitize 808 nm light and releases red luminescence, which affects spectral purity. As a result, a series of Core@S1@S2@S3@S4 samples with different thickness of S3 shell (t = 7.4 nm, 8.7 nm and 14.1 nm) are synthesized (Figure 3B). Under excitation by 808 nm light, the Nd^3+^ in the S3 layer absorb the excitation light and transfer the energy to the Yb^3+^ ions and then Ho^3+^ activators, via the energy transfer pathway, i.e., 808 nm→Nd→Yb→Ho. The green emission arises from the transition of (^5^S_2_, ^5^F_4_)→^5^I_8_ of Ho^3+^ ions, which dominates the emission color of Ho^3+^ ions (Figure S4A). However, the pre-existing S1 shell (NaErF_4_:Tm) can also sensitize 808 nm light and releases red luminescence, which affects spectral purity. Therefore, as shown in Figure S4B, the green to red (G/R ratio) intensity ratio is only 0.7 upon 808 nm light excitation when the thickness of S3 is about 7.4 nm. As the thickness of S3 shell is increased, the G/R ratio value is accordingly increased. The green luminescence already dominates the upconversion emission when the thickness of S3 shell reaches 14.1 nm. This is because the S3 shell acts as a filter and it can prevent the 808 nm light from reaching the inner S1 shell.

As for the blue-emitting portion, a series of Core@S1@S2@S3@S4@S5 nanoparticles with different thickness of S5 shell are investigated (Figure 3C). Upon excitation by 980 nm light, Yb^3+^ ions in the S5 shell absorb the excitation light and transfer the energy to Tm^3+^ activator ions. The major emission peaks at around 450 and 475 nm can be assigned to the ^1^D_2_ → ^3^F_4_, ^1^G_4_ →^3^H_6_ transitions of Tm^3+^ ions (Figure S5A). Notably, the 980 nm light can also be absorbed by the Yb^3+^-Ho^3+^ ions in the S3 shell, resulting in green emission. For example, the blue to green intensity ratio (B/R ratio) is only about 0.34 upon excitation by 980 nm light when the thickness of S5 layer is only about 4.4 nm (Figure S5B). However, as the thickness of S5 shell is increased, the Yb^3+^-Tm^3+^ pair can act as a photon filter and significantly deplete the 980 nm light, inhibiting the inward propagation of incident light. As a result, by carefully controlling the thickness of the shell 5 layer, the dominant blue emission of Tm^3+^ ions can be acquired.

Next, we studied the emissive profiles of as-prepared core-multi-shell UCNPs at NIR lights with different power densities (Figures 3D–3F). It shows that the red, green, and blue emissions enhance gradually with increasing the excitation power of 1550 nm, 808 nm, and 980 nm light accordingly. Besides, the line fitting results indicate that there is a three-photon absorption process under 1550 nm and 980 nm excitation, and a two-photon absorption process under 808 nm excitation (Figure S6).

### Synthesis of mesoporous-silica-coated UCNPs

**Figure 4:**
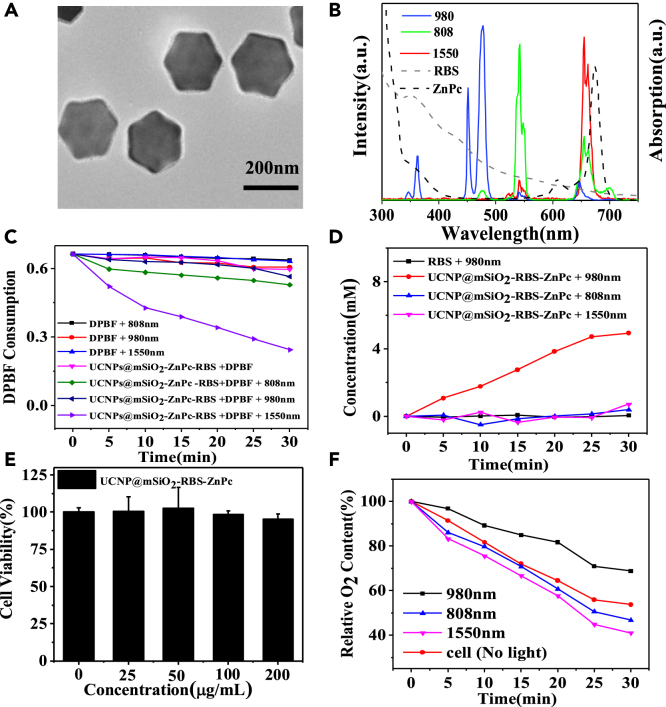
Characterization and ROS release of UCNP-based therapeutic agent (A) TEM image of UCNPs@mSiO_2_. (B) Upconversion luminescence spectra of UCNPs@mSiO_2_ under excitation at 808 nm, 980 nm, 1550 nm laser light and the absorption spectra of ZnPc and RBS. (C and D) Comparison of DPBF consumption (C) and NO release (D) of different treatment groups. (E) Cell viability of HeLa cells incubated with UCNPs@mSiO_2_-ZnPc-RBS solution at different concentrations. (F) Relative O_2_ content of HeLa cells under different treatment conditions in a hypoxic environment.C.

The as-synthesized UCNPs usually contain hydrophobic ligands, such as oleic acid, which restrict their biological applications. In order to improve the water solubility and biocompatibility, mesoporous silica (mSiO_2_) is coated on the surface of UCNPs (UCNPs@mSiO_2_). As shown in Figure 4A, a uniform mesoporous layer (∼5 nm thick) is decorated on the surface of UCNPs. According to the N_2_ adsorption/desorption isotherm curve, the surface area of UCNPs@mSiO_2_ is calculated to be 679 m^2^ g^−1^ and the average pore size is about 4.32 nm (Figure S7).

### Construction of UCNPs@mSiO_2_-ZnPc-RBS nanoplatform

Figure 4B shows the upconversion luminescence spectra of UCNPs@mSiO_2_ under different NIR laser light excitations. Similar to the core UCNPs, UCNPs@mSiO_2_ nanocomposite still enables to emit green, blue, and red luminescence under 808 nm, 980 nm, and 1550 nm laser light, respectively. Subsequently, two types of photosensitizer drugs, i.e., zinc phthalocyanine (ZnPc) and RBS salt were loaded into porous channels of UCNPs@mSiO_2_ nanocomposite in order to construct a programmable therapeutic nanoplatform (UCNPs@mSiO_2_-ZnPc-RBS). The reason why ZnPc photosensitizer is chosen as the ^1^O_2_ generator for PDT treatment is because it has a strong absorption in the spectral region of 600–700 nm (the black dashed line in Figure 4B), which well matches the red light emission of UCNPs@mSiO_2_ under 1550 nm excitation. Meanwhile, RBS salt is used as the NO donor because its absorption spectrum (the gray dashed line) overlaps with the ultraviolet emission (< 400 nm) of UCNPs@mSiO_2_ under 980 nm excitation. As a result, the specially designed UCNPs@mSiO_2_-ZnPc-RBS nanocomposite can independently release NO and ROS in response to two different excitation lights, which ensures the occurrence of different photoactivations of therapeutic at the right place and right time.

### Independent release of ROS and NO in solution

First, 1,3-diphenylisobenzofuran (DPBF) dye was used as a probe to detect the generation of ROS because its absorbance at around 417 nm could be specifically decreased by ROS. Figure 4C shows the absorbance changes of DPBF after 30 min of NIR light under different conditions. It can be observed that the absorption peak of DPBF drops rapidly only under 1550 nm light excitation while no significant absorbance changes of DPBF dye under either 980 nm or 808 nm light excitations, indicating that ROS signals are mainly produced by UCNPs@mSiO_2_-ZnPc-RBS nanocomposites under 1550 nm light excitation. Griess analysis kit method was then used to probe the production of NO of UCNPs@mSiO_2_-ZnPc-RBS under different irradiation conditions, as shown in Figure 4D. Under 980 nm light, NO signal is observed to be steadily increased while little NO is produced under 1550 nm or 808 nm light excitation. Clearly, the results in Figures 4C and 4D show that the release of ROS and NO can only be activated by 1550 nm and 980 nm light, respectively. The independent control of ROS and NO generation is quite important as it offers a convenient tool for sequential photoactivation of NO and ROS release, which is also critical for programmed therapeutic applications.

### Programmed therapeutic processes *in vitro*

Prior to use for therapeutic applications, it is necessary to test the cytotoxicity of UCNPs@mSiO_2_-ZnPc-RBS nanocomposites. HeLa cells were incubated with UCNPs@mSiO_2_-ZnPc-RBS at different concentrations (0, 50, 100, and 200 μg/mL) for 24 h, and the cell viability was evaluated using the CCK-8 assay. As shown in Figure 4E, the cell viability does not change significantly as the concentration of UCNPs@mSiO_2_-ZnPc-RBS increases, indicating good biocompability of as-developed UCNPs@mSiO_2_-ZnPc-RBS nanocomposites. Additionally, confocal laser scanning microscope (CLSM) was used to image the intracellular uptake of UCNPs@mSiO_2_-ZnPc-RBS nanocomposites. Lyso-Tracker and Mito-Tracker dye was used to stain lysosomes and mitochondria, respectively, while green luminescence upon 808 nm light illumination was used to collect the signal of UCNPs@mSiO_2_-ZnPc-RBS nanocomposites. The upconversion luminescence of UCNPs@mSiO_2_-ZnPc-RBS gradually overlaps with lysosome (Figure S8A) and mitochondria (Figure S8B) signals as the incubation time increases. As shown in Figure S8C, the Pearson’s correlation coefficient for the UCNPs and lysosomes is continuously increased from 0.10 after 2 h of incubation to 0.48 after 24 h of incubation. Similar increase of Pearson’s correlation coefficient can also be observed between UCNPs and mitochondria (Figure S8D). These results clearly show that the as-prepared UCNPs@mSiO_2_-ZnPc-RBS nanocomposites can be effectively taken up by HeLa cells.

Next, the oxygen consumption in the cell culture was measured in order to determine the role of NO in inhibiting cellular respiration activities. Experimentally, HeLa cells were first incubated with UCNPs@mSiO_2_-ZnPc-RBS for 24 h in the dark, which were then treated by 808 nm, 980 nm, and 1550 nm light (1 W/cm^2^,10 min), respectively. Figure 4F presents the measured oxygen content in the cell culture, which explicitly demonstrates that the culture medium illuminated by 980 nm light has the highest oxygen content. It is believed that the NO released upon 980 nm light can inhibit the mitochondrial respiratory chain,^43^ thus reducing oxygen consumption. It should be noted that the inhibitory control of NO on mitochondrial respiration is quite important as more oxygen can be saved to combat the hypoxia environment in the tumor region and accordingly promote the generation of ROS in the PDT modality.

**Figure 5:**
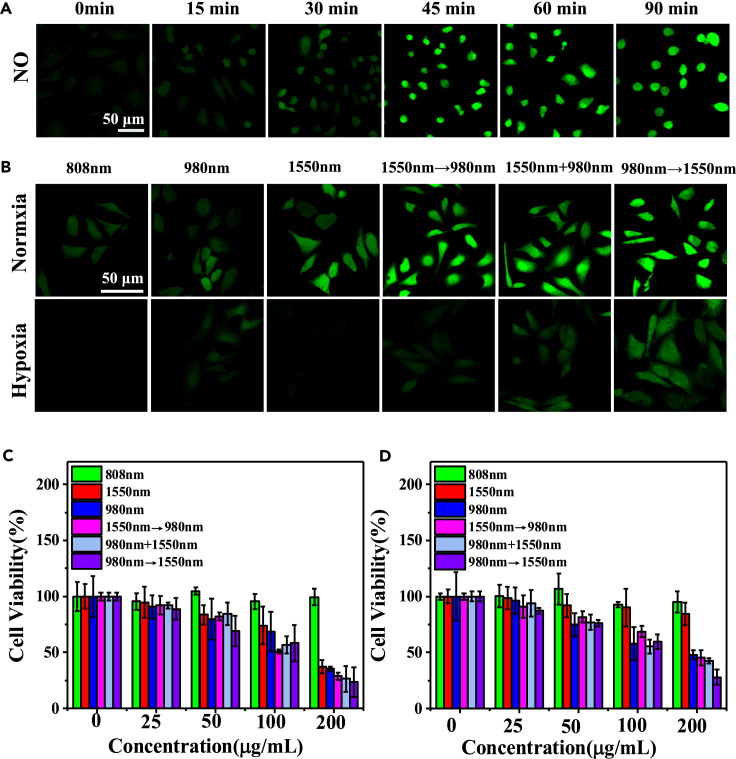
Intracellular NO and ROS imaging, cell viability with UCNPs@mSiO_2_-ZnPc-RBS in varied oxygen conditions (A) Confocal imaging of intracellular NO production at different times after irradiation by 980 nm light. (B) Comparison of intracellular ROS under normoxic and hypoxic conditions. (C and D) Cell viability of HeLa cells cultured with different concentrations of UCNPs@mSiO_2_-ZnPc-RBS under different treatment conditions in normoxia (C) and hypoxia (D).

In order to further verify the generation of NO intracellularly, HeLa cells were cultured with UCNPs@mSiO_2_-ZnPc-RBS nanocomposites, illuminated by the 980 nm light (1 W/cm^2^) for 10 min, and then imaged using CLSM at 0 min, 15 min, 30 min, 45 min, 60 min, and 90 min after the 980 nm light was turned off. From Figure 5A, it can be seen that the intracellular NO signal gradually increases over time. Particularly, the NO signal within 30 min is still relatively small while adequate amount of NO can be observed at 60 min after illumination. These results demonstrate that it takes time for the NO to be efficiently released and distributed inside cells, stressing the necessity of step-by-step activation of NO and ROS release. This is because if NO and ROS are released at the same time, NO may have limited time to bind to the mitochondria and the generation of ROS may still be hampered in the hypoxic environment. Figure 5B compares the intracellular ROS signals under different excitation conditions in hypoxia and normoxia environments. As expected, it is the highest using the sequence of 980 nm→1550 nm (980 nm light first and then 1550 nm light) under hypoxia condition. The quantitative analysis of the luminescence intensity further confirms that the ROS intensity produced under hypoxia environment via the sequence of 980 nm→1550 nm is about 2 times stronger than that of the 1550 nm→980 nm (1550 nm light first and then 980 nm), and about 2.7 times than the 1550 nm + 980 nm (1550 nm and 980 nm light at the same time).

Next, the influence of NO release on the cellular function is investigated. Specifically, a probe of 5,5′,6,6′-tetrachloro-1,1′,3,3′-tetraethylbenzimidazolo-carbocyanine iodide (JC-1) was used to evaluate the mitochondrial membrane potential after NO treatment. In general, JC-1 exists in two forms: JC-1 monomer and JC-1 aggregate. In healthy cells, JC-1 enters the mitochondrial and forms JC-1 aggregates, emitting strong red color. While in unhealthy or apoptotic cells, JC-1 remains in the cytoplasm in its monomeric form, displaying green color. As shown in Figure S9A, for the NO-treated cells, strong green signals can be observed, in a comparison with the control group. This clearly indicates that the release of NO under excitation by 980 nm light can lead to the depolarization mitochondrial membrane potential and cause mitochondrial damage. In light of the influence of NO and mitochondria function, we have then evaluated the generation of ATP (adenosine triphosphate) after NO treatment. Figure S9B indicates that, the NO treatment, via excitation by 980 nm light, results in much lower ATP content compared with groups treated with PBS and no light excitation. This is because the release of NO depolarizes mitochondrial membrane and reduces the membrane potential, which consequently affects cellular respiration and diminishes ATP production. Additionally, the intracellular oxygen consumption is closely related with the activity of an oxidase, called cytochrome *c* oxidase (CcO), which can convert oxygen into water through the respiration chain. Therefore, the CcO assay is then used to evaluate its activity. As shown in Figure S9C, the CcO activity is significantly suppressed in cells treated with NO (UCNPs@mSiO2-ZnPc-RBS+980 nm light) compared to the other two groups (PBS and UCNPs@mSiO_2_-ZnPc-RBS). This is because the released NO can bind to the CcO and affect its activity, thereby inhibiting cellular respiration.^44^^,^^45^

The specially developed programmable activation of phototherapeutics was then evaluated based on the cell viability *in vitro*. HeLa cells were first incubated with UCNPs@mSiO_2_-ZnPc-RBS at different concentrations (0, 50, 100, and 200 μg/mL) and then treated by 808 nm light, 980 nm light, 1550 nm light, and their different combinations under normoxia (Figure 5C) and hypoxia conditions (Figure 5D).

As shown in Figures 5C and 5D, the cell viability of HeLa cells treated with 808 light excitation can still remain around 100%, indicating that the 808 nm light has negligible influence on the cell viability. Therefore, the green emission released upon 808 nm light illumination can be used as an imaging tool for diagnostic purpose. For the cells treated with 980 nm light, the generated nitric oxide (NO) can disturb cell metabolism and lead to oxidative stress destruction and free radicals. As a result, the reduction of cell viability can be observed in both normoxia and hypoxia environments. Upon illumination by 1550 nm light only, the cell viability is found to be below 40% under normoxia condition when HeLa cells were cultured with 200 μg/mL of UCNPs@mSiO_2_-ZnPc-RBS, indicating adequate amount of ROS is generated during the PDT process. However, the cell viability is increased to around 50% under hypoxia condition. Similar results are also observed for the cells treated with 1550 nm light first and then 980 nm (1550 nm→980 nm), and with 1550 nm and 980 nm light at the same time (980 nm + 1550 nm). The increase of cell viability in the hypoxia environment can be attributed to the reduction of oxygen content and accordingly less generation of ROS. Conversely, for the HeLa cells treated using 980 nm light first and then 1550 nm light (980nm→1550 nm), the cell viability is only slightly increased from 23% under normoxia condition to 28% under hypoxia condition. Furthermore, the same trend is also consistent with the quantitative analysis of flow cytometry (Figure S10). Notably, the establishment of hypoxia condition is mainly used to mimic the oxygen-deficient environment in the tumor region. It is rather clear that the ROS generation in PDT is greatly affected by the oxygen presence in the surrounding environment. Under this circumstance, the programmable approach, 980 nm→1550 nm, has demonstrated outstanding performance in combating the hypoxia obstacle. Upon excitation by 980 nm light, NO is firstly released, which can then bind to the mitochondria and hence inhibit the cellular respiration. Therefore, more oxygen can be saved for the generation of ROS in PDT activated by the subsequent 1550 nm light.

### Programmed phototherapy *in vivo*

For the programmed phototherapy *in vivo*, the female nude mouse was used as an animal model. First, the mice were subcutaneously injected with 4T1 tumor cells.

**Figure 6:**
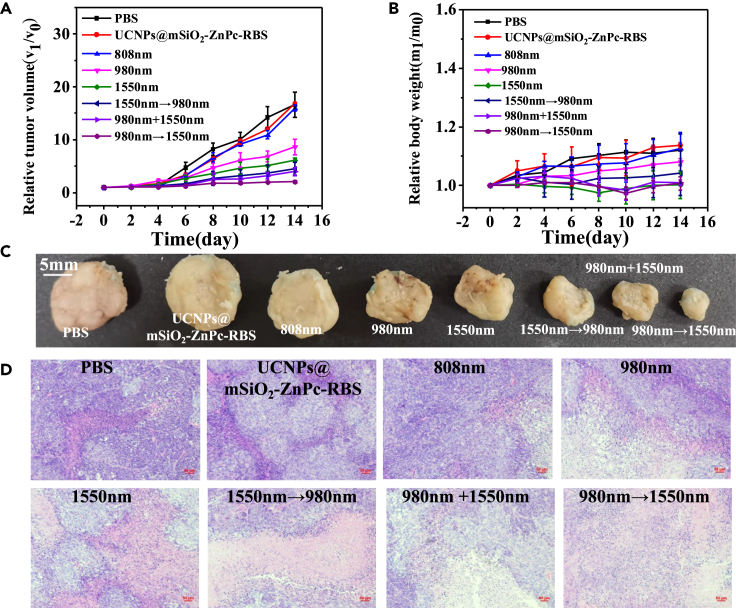
*In vivo* study of UCNPs@mSiO_2_-ZnPc-RBS (A and B) Relative tumor volume (A) and body weight (B) in different groups after different treatments. (C) The photographs of segregated tumors after treatment. (D) H&E stained images of tumors after treatment for 14 days.

Although the internalization of UCNPs within living organisms depends on their size and surface ligands, the UCNPs with the size range of 50∼250 nm are internalized via endocytosis mechanism.^46^ Once inside the cells, UCNPs may rapidly undergo morphological transformation in the physiological environment, such as the acidic medium of lysosomes. Another previous study has also reported that UCNPs may be decomposed (dissolved) and excreted via feces and urine.^47^ As a result, the UCNP-based solution is injected into the mouse every three days during the treatment. When the tumor grew to about 100 mm^3^ in size, the mice were randomly divided into eight groups with five mice in each group: control group 1 (PBS solution), control group 2 (UCNPs@mSiO_2_-ZnPc-RBS), group 3 (808 nm), group 4 (980 nm), group 5 (1550 nm), and group 6 (1550 nm→980 nm), group 7 (1550 nm + 980 nm), and group 8 (980 nm→1550 nm). The laser power density is 1 W/cm^2^, and the illumination time for each group is 20 min in total. During the 14 days of treatment, the body weight and tumor size were measured every other day. Figure 6A compares the growth of tumors in different groups after treatment. It can be seen that the tumors in the control group, group 1, group 2 and group 3 grow rapidly, while the growth of tumors in groups 4, 5, 6, and 7 is slightly inhibited. In contrast, the tumor size in group 8 is the smallest, indicating the tumor growth is greatly inhibited by the programmed phototherapy under the sequence of scheduling NO release first, and then ROS.

Figure 6B shows the changes of relative body weight in eight groups within 14 days. NO significant decrease or increase in the body weight is observed, indicating that the as-developed nanocomposites have good biocompatibility. After 14 days of treatment, the mice in all groups were sacrificed and the tumor tissues were stripped. As shown in Figure 6C, the tumor volume is the smallest for the mice group treated with the light illumination in the sequence of 980 nm→1550 nm. In order to further prove the therapeutic effect, we also performed histological hematoxylin-eosin staining (H&E) staining analysis on different groups of tumors and major organs. Figure 6D presents the H&E stained images of tumor tissue sections. Compared with other groups, most severe apoptosis or necrosis and nucleus contraction are also observed in the group of 980 nm→1550 nm Figure S11 shows the H&E stained images of main organs (heart, liver, spleen, lung, and kidney) of mice in different groups. No evident damages are observed, which indicates that the as-prepared UCNPs@ mSiO_2_-ZnPc-RBS has no short-term toxicity and demonstrates great potential for clinical applications.

## Discussion

Due to their deeper tissue penetration depth and unique optical properties, lanthanide-doped UCNPs have been widely used as light transducers to excite photosensitizers for many types of phototherapeutic applications, such as PDT, gas therapy, chemotherapy, and their combinational therapies.^48^^,^^49^^,^^50^^,^^51^ Taking PDT as an example, UCNPs have been widely employed to activate PDT photosensitizers for release of ROS since the first reported study.^52^ As for the gas therapy, application of UCNPs to trigger NO donors to generate NO was initially demonstrated by Zhang et al. in 2015.^53^ A summary of the progress of NO-involved therapy based on UCNP nanocomposites is presented in Table S1. It indicates that NO has been used to alleviate tumor hypoxia or overcome multidrug resistance in combination with PDT or chemotherapy. Moreover, NO can trigger the immune response for NO-activated immunotherapy or enhance radiotherapy as an effective hypoxic radiosensitizer. However, in previous studies, NO and other therapeutic molecules (e.g., ROS and DOX (doxorubicin)), are mainly released simultaneously rather than in a programmable manner during the photoactivation process. For an ideal phototherapy treatment, it should be methodically optimized and each of its key processes should be controlled in a sequential manner, which means that appropriate photoactivation should be administered at the appropriate time and for the appropriate duration. However, such level of accuracy in UCNP-based phototherapy is limited because previously reported UCNPs do not allow for separate photoactivation of different therapeutic processes, as they are commonly responsive to only one excitation wavelength.

One approach to address this issue is to develop orthogonal emissive UCNPs that can emit different colors in response to different excitation lights. For example, these orthogonal emissive UCNPs have been used for cell imaging and phototherapy independently.^11^ However, in these studies, the UCNPs can only respond to two NIR excitation lights and programmable control over more complicated processes is still lacking. The active-core/active shell approach has been successfully applied to tune the upconversion luminescence in lanthanide-doped nanoparticles.^54^ In this study, we have employed an energy segmentation-based strategy to construct core-multi-shell structured UCNPs that enable to release orthogonal trichromatic colors in response to three NIR lights. Because more excitation and emission profiles are independently controlled, the as-developed UCNP-based nanoplatform allows for spatiotemporal control over more than two processes in a PDT treatment, including imaging diagnostics, suppression of tumor hypoxia and release of ROS. As a result, the treatment efficiency is significantly enhanced via such a stepwise manner. It is believed that the as-reported nanoplatform represents a high-level precision of phototherapy and would be useful for programming and customized cancer treatment.

### Conclusions

In this study, orthogonal emissive UCNPs, NaYF_4_@NaErF_4_:Tm@NaYF_4_@NaYF_4_:Yb,Ho,Nd@NaYF_4_@NaYF_4_:Yb,Tm, are reported. By precisely tuning the thickness of luminescent and inert shells, the as-developed UCNPs can release orthogonal trichromatic colors in response to three NIR lights, i.e., red emission upon 1550 nm light excitation, green emission upon 808 nm light excitation, and blue emission upon 980 nm light excitation. As a proof of concept, these photoswitchable UCNPs are further incorporated with the photosensitizer (Zinc phthalocyanine, ZnPc) and NO donor (RBS to construct an intelligent “off-on” theranostic PDT nanoagent. Thanks to the orthogonal emissive properties of UCNPs, imaging guidance, NO generation, and ROS release can be triggered independently upon alternating irradiation with 808 nm light, 980 nm light, and 1550 nm light, allowing for a cascaded therapeutic modality. The results demonstrate that the sequential activation of NO and PDT can dramatically alleviate tumor hypoxia and greatly enhance the PDT efficiency, which is believed to have a new avenue for future clinical phototherapeutic treatment.

### Limitations of the study

This study uses an energy segmentation-based strategy to synthesize trichromatic UCNPs. A notable limitation lies in the requirement for five shells to be applied onto the core nanoparticle to achieve distinct and orthogonal emissions using different excitation lights. Clearly, this makes the fabrication rather challenging and time-consuming. Future research could explore alternative and more convenient strategies to simplify the nanoarchitecture by using fewer shells.

Besides, the phototherapeutic process during the PDT treatment does not only involve the consumption of oxygen and generation of ROS. As a result, more detailed investigation is currently underway to study the PDT treatment on the biochemical level, thereby enhancing the understanding of its underlying mechanisms.

## STAR★Methods

### Key resources table

**Table:** 

REAGENT or RESOURCE	SOURCE	IDENTIFIER
**Chemicals, peptides, and recombinant proteins**		

ErCl_3_	Sigma-Aldrich	CAS#: 10025-75-9
YbCl_3_	Sigma-Aldrich	CAS#: 10361-92-9
YCl_3_	Sigma-Aldrich	CAS#: 10361-92-9
NdCl_3_	Sigma-Aldrich	CAS#: 10024-93-8
NaOH	Sinopharm	CAS#: 1310-73-2
NH_4_F	Sinopharm	CAS#: 12125-01-8
C_18_H_36_	Sigma-Aldrich	CAS#: 593-45-3
C_18_H_34_O_2_	Sigma-Aldrich	CAS#: 1957-11-04
CH_3_CH_2_OH	Tansoole	CAS#: 64-17-5
C_6_H_12_	Tansoole	CAS#: 110-82-7
CH_3_OH	Tansoole	CAS#: 67-56-1
CTAB	Sigma-Aldrich	CAS#: 57-09-0
TEOS	Sigma-Aldrich	CAS#:78-10-4
DPBF	Sigma-Aldrich	CAS#: 5471-63-6
CCK-8 kit	Beyotime	N/A
DCFA-DH	Beyotime	N/A
Lyso-tracker-Green	Beyotime	N/A
Mito-Tracker-Red	Beyotime	N/A

**Experimental models: Cell lines**		

HeLa	Shanghai University	N/A

**Experimental models: Organisms/strains**		

Female nude mice	Shanghai Laboratory Animal Center	N/A

### Resource availability

#### Lead contact

Further information and requests for resources should be directed to and will be fulfilled by the lead contact, Xiaohui Zhu (xhzhu@shu.edu.cn).

#### Materials availability

No new materials were created during this study.

### Experimental model and study participant details

#### Animals and tumor models

Female nude mice (4–5 weeks old) were provided by the Shanghai Laboratory Animal Center (SLAC, Shanghai). In order to build a *in vivo* tumor model, 4T1 tumor cells were subcutaneously injected into the mice. When the tumors grew to about 100 mm^3^ in size, the *in vivo* treatment was conducted. All animal-related experiments were performed with the guidelines of the Institutional Animal Care and Use Committee of Shanghai University.

#### Cell culture

Human cervical cancer cells (HeLa cell) were cultured in DMEM medium containing 10% FBS (fetal bovine serum), penicillin (100 U/mL), and streptomycin (100 μg/mL) at 37°C incubator. HeLa cells were seeded in small dishes at a density of 1.5×10^5^ cells per dish for 24 h and incubated with samples for 24 h subsequently.

### Method details

#### Preparation of core-multi-shell UCNPs

##### Step 1: Synthesis of the core nanoparticles

1.0 mmol YCl_3_ aqueous solution was added to a 100 mL three-necked flask, and the temperature was raised to 120°C. Then, 6 mL oleic acid (OA) and 15 mL 1-octadecene (ODE) were added and heated to 150°C with constant stirring for 30 min to form the lanthanide–oleic acid complex, which was then cooled down to 60°C. 8 mL methanol solution containing 100 mg NaOH and 150 mg NH_4_F was added to the flask and heated to 120°C for 35 min. Thereafter, the temperature was rapidly increased to 300°C under Ar atmosphere for 1 h. After the reaction was complete, the nanoparticles were washed with cyclohexane/ethanol several times. Finally, the products were stored in 20 mL cyclohexane.

##### Step 2: Synthesis of core-shell UCNPs

0.199 mmol ErCl_3_ and 0.001 mmol TmCl_3_ were added to 100 mL three flasks. Then, the temperature was raised to 120°C. 6 mL oleic acid (OA) and 15 mL 1-octadecene (ODE) were added and heated to 150°C for 30 min. Next, cyclohexane solution containing 40 mg core nanoparticles was injected into the flask, kept at 150°C to remove cyclohexane and then cooled down to 60°C. 8 mL methanol solution containing 50 mg NaOH and 75 mg NH_4_F were added to the flask and heated to 120°C for 35 min. Thereafter, the temperature was rapidly increased to 300°C under Ar atmosphere for 1 h to grow core-shell nanoparticles. After the reaction was complete, the nanoparticles were washed with cyclohexane/ethanol several times and redispersed in cyclohexane.

##### Step 3: Synthesis of core-shell-shell UCNPs

The synthesis of NaYF_4_@NaErF_4_:Tm@NaYF_4_(Core@S1@S2) was similar to that of Core@S1 nanoparticles, except that the Core@S1 nanoparticles were used as seeds while YCl_3_ was used as the shell precursor. Besides, the thickness of S2 shell could be varied by controlling the amount of YCl_3_ precursor.

##### Step 4: Synthesis of core-shell-shell-shell UCNPs

The synthesis of NaYF_4_@NaErF_4_:Tm@NaYF_4_@NaYF_4_:Yb,Ho,Nd (Core@S1@S2@S3) synthesis was similar to that of Core@S1@S2 nanoparticles, except the Core@S1@S2 nanoparticles were used as seeds while YCl_3_, YbCl_3_, HoCl_3_, and NdCl_3_ were used as shell precursor.

##### Step 5: Synthesis of core-shell-shell-shell-shell UCNPs

The synthesis of NaYF_4_@NaErF_4_:Tm@NaYF_4_@NaYF_4_:Yb,Ho,Nd @NaYF_4_ (Core@S1@S2@S3@S4) was similar to that of Core@S1@S2@S3 nanoparticles, except that the Core@S1@S2@S3 nanoparticles were served as seeds while YCl_3_ was used as the S4 precursor.

##### Step 6: Synthesis of core-shell-shell-shell-shell-shell UCNPs

The synthesis of NaYF_4_@NaErF_4_:Tm@NaYF_4_@NaYF_4_:Yb,Ho,Nd @NaYF_4_ @NaYF_4_:Yb,Tm (Core@S1@S2@S3@S4@S5) was similar to that of Core@S1@S2@S3@S4 nanoparticles, except that the Core@S1@S2@S3@S4 nanoparticles were served as seeds while YCl_3_, YbCl_3_, and TmCl_3_ was used as the S5 precursor.

#### Preparation of mesoporous silicon modified UCNPs (UCNPs@mSiO_2_)

Firstly, 3 mL as-prepared UCNPs (approximately 45 mg) was added to 20 mL ultra-pure water containing 0.3 g CTAB. The solution was then vigorously stirred overnight to evaporate the cyclohexane. Next, 40 mL distilled water, 6 mL ethanol, 300 μL NaOH (2 M) was added successively to a 250 mL three-neck flask and heated to 70°C stirring for 30 min. Thereafter, 400 μL TEOS was slowly added drop by drop under vigorous magnetic stirring. The reaction was cooled down to room temperature. The products were centrifuged and washed 3–4 times with ethanol. In order to remove the CTAB template, the precipitate was dissolved in 40 mL acidic ethanol solution (pH∼1.5) and stirred at 70°C for 3 h. The above etching process was repeated three times and the obtained precipitate, UCNPs@mSiO_2_, were finally stored in 10 mL ethanol.

#### Loading ZnPc and RBS photosensitizers into UCNPs@mSiO_2_

5 mg UCNPs@mSiO_2_, 1.2 mg ZnPc and 1.2 mg RBS were dissolved in 2 mL pyridine solution and stirred for 12 h under dark conditions. The precipitate was centrifuged and washed three times with deionized water. The obtained product (UCNPs@mSiO_2_-ZnPc-RBS) is dispersed in deionized water.

#### Detection of ROS

1,3-diphenylisobenzofuran (DPBF) probe was used to detect the ability of UCNPs@mSiO_2_-ZnPc to release ROS. Under dark conditions, 10 μL ethanol solution of DPBF (10 mM) was mixed with 2 mL UCNPs@mSiO_2_-ZnPc-RBS solution. The solution was then irradiated with 808 nm, 980 nm and 1550 nm lasers for 30 min, respectively. The UV-Vis absorption of DPBF was tested every 5 min. Meanwhile, control experiments were also performed in the pure DPBF and laser-free irradiation groups.

The generation of intracellular ROS was performed by using DCFH-DA as a probe. Human cervical cancer cells (HeLa cell) were cultured in DMEM medium containing 10% FBS (fetal bovine serum), penicillin (100 U/mL), and streptomycin (100 μg/mL) at 37°C incubator. HeLa cells were seeded in small dishes at a density of 1.5×10^5^ cells per dish for 24 h and incubated with UCNPs@mSiO_2_-ZnPc-RBS (2 mL) for 24 h subsequently. The DCFH-DA probe was added to small dishes and placed into an incubator for 30 min, and cells were washed multiple times with PBS. The small dishes were then placed in or not in a sealed box with anaerobic gas producing bags (5% CO_2_ and less than 1% O_2_) and incubated for 1 h to mimic normoxic or hypoxia conditions in the dark. Cells in the corresponding small dishes were irradiated by 808 nm light (1 W/cm^2^,10 min), 980 nm (1 W/cm^2^,10 min), 1550 nm (1 W/cm^2^,10 min), and their combinations with different sequences (980 nm + 1550 nm, 980 nm→1550 nm, and 1550 nm→980 nm). Notably, 980 nm + 1550 nm means that cells were irradiated by 980 nm and 1550 nm for 10 min simultaneously, 980 nm→1550 nm means that cells were firstly irradiated with 980 nm for 5 min, followed by 1550 nm for another 5 min, and 1550 nm→980 nm means that cells were firstly irradiated with 1550 nm for 5 min, followed by 980 nm for another 5 min.

#### Detection of nitric oxide

A commercially available Griess detection kit was used to detect NO. UCNPs@mSiO_2_-ZnPc-RBS containing solutions were irradiated with 808 nm, 980 nm and 1550 nm light for 30 min, respectively. Then, 200 μL of suspension was extracted every 5 min, and the supernatant was retained by centrifugation. Griess reagent I and Griess reagent II were then added. Whereafter, the TecanSpark multifunctional microplate reader was used to measure the fluorescence intensity of the mixed solution at a wavelength of 540 nm. Meanwhile, the non-laser irradiation group was used as a control experiment. Intracellular NO was detected using a commercial DAF-FMDA fluorescence probe. HeLa cells were first incubated with UCNPs@mSiO_2_-ZnPc-RBS for 24 h, stained with DAF-FMDA for 30 min. Then, the cells were treated by 980 nm laser irradiation (1 W/cm^2^, 10 min) and then observed under a CLAM at 0,15,30,45,60, and 90 min after irradiating.

#### Measurement of oxygen content in cell culture

HeLa cells were seeded in small dishes at a density of 1.5 × 10^5^ cells per dish for 24 h and incubated with UCNPs@mSiO_2_-ZnPc-RBS (2 mL) for 24 h. Small dishes were placed in an anaerobic nitrogen glove box and then irradiated with 808 nm, 980 nm and 1550 nm (1 W/cm^2^,10 min) laser, respectively. After that, the oxygen content was recorded every 5 min using oxygen electrodes. The relative oxygen content was calculated as the ratio of the oxygen content measured at each time point to that measured at the initial stage.

#### Confocal imaging of cellular uptake of UCNPs@mSiO_2_-ZnPc-RBS

HeLa cells were seeded in small dishes at a density of 1.5 × 10^5^ cells per dish for 24 h and incubated with UCNPs@mSiO_2_-ZnPc-RBS (2 mL) for 2 h, 8 h, 12 h, and 24 h, respectively. Lyso-Tracker-Green and Mito-Tracker-Red dye were added to the cells to stain the lysosome and mitochondria. Then, cells were washed multiple times with PBS and cellular uptake imaging was performed using a confocal laser scanning microscope.

#### Evaluation of apoptosis

HeLa cells were seeded in a 6-well culture plate at a density of 1.5 × 10^5^ cells per well for 24 h, and incubated with UCNPs@mSiO_2_-ZnPc-RBS for 24 h. The 6-well plate was then placed in or not in a sealed box containing anaerobic gas-producing bags (5% CO_2_and less than1% O_2_) to simulate hypoxia or normoxic conditions in the dark. Cells in the corresponding small dishes were respectively irradiated with 808 nm, 980 nm, 1550 nm, 980 nm→1550 nm, 1550 nm→980 nm and 980 nm + 1550 nm (1 W/cm^2^,10 min) laser light, and then incubated in the incubator for another 4 h. After that, cells were transferred into a centrifuge tube, centrifuged at 900 r/min for 5 min and washed twice by PBS. The cells were added into a 500 μL of buffer, stained using Annexin V-FITC and PI for 20 min under dark conditions, and then measured by flow cytometry.

#### Assessment of cytotoxicity and *in vitro* PDT treatment

HeLa cells were seeded in a 96-well culture plate at a density of 8000 cells per well for 24 h. A series of culture medium solutions with 100 μL UCNPs@mSiO_2_-ZnPc-RBS (0, 50, 100, and 200 μg/mL) were added to cell well plates for 24 h. Then, the CCK-8 assay was used to evaluate the cell viability. For the *in vitro* PDT experiment, the cells were incubated with different concentrations of UCNPs@mSiO_2_-ZnPc-RBS for 24 h in 96-well plates. The 96-well plate was then placed in or not in a sealed box containing anaerobic gas-producing bags (5% CO_2_ and less than 1% O_2_) to simulate hypoxia or normoxic condition. Cells in the corresponding 96-well plates were respectively irradiated with 808 nm, 980 nm, 1550 nm, 980 nm→1550 nm, 1550 nm→980 nm and 980 nm + 1550 nm (1 W/cm^2^,10 min) laser light, and then incubated in the incubator for another 24 h. 10 μL CCK-8 reagent was subsequently added to each well plate and incubated in the incubator for another 2 h. The absorbance at 450 nm was recorded in a Tecan Spark multifunctional microplate reader.

#### *In vivo* PDT/ NO therapy experiments in mice

First, mice were injected with 4T1 tumors subcutaneously. When the tumors grew to about 100 mm^3^ in size, the mice were randomly divided into eight groups with five mice in each group: control group 1 (PBS solution), control group 2 (UCNPs@mSiO_2_-ZnPc-RBS), group 3 (UCNPs@mSiO_2_-ZnPc-RBS with 808 nm), group 4 (UCNPs@mSiO_2_-ZnPc-RBS with 980 nm), group 5 (UCNPs@mSiO_2_-ZnPc-RBS with 1550 nm), and group 6 (UCNPs@mSiO_2_-ZnPc-RBS with 1550 nm＋980 nm), group 7 (UCNPs@mSiO_2_-ZnPc-RBS with 1550 nm→980 nm), and group 8 (UCNPs@mSiO_2_-ZnPc-RBS with 980 nm→1550 nm). The laser power density is 1 W/cm^2^, and the illumination time for each group is 20 min in total. The body weight and tumor size were measured every other day within the 14 days of treatment. Relative body weight was calculated as m/m_0_, where m_0_ and m indicates body weight before and after treatment, respectively. Tumor volume is calculated as follows: Tumor volume (v)= (length and width^2^)/2. Relative tumor volume was calculated as v/v_0_ (where v_0_ is the tumor volume at the beginning of treatment). After 14 days of treatment, the main organs (heart, liver, spleen, lung and kidney) and tumor tissues of the eight groups of mice were H&E stained and imaged by fluorescence microscopy.

### Quantification and statistical analysis

Standard error of the mean is shown on figures unless otherwise noted. The study does not include any specific quantification and statistical analysis.

## Data Availability

• Data reported in this paper will be shared by the lead contact upon request. • This paper does not report original code. • Any additional information required to reanalyze the data reported in this work paper is available from the lead contact upon request

## References

[1] Huang Z. (2005). A review of progress in clinical photodynamic therapy. Technol. Cancer Res. Treat..

[2] Wu X., Peterson R.B., Gadde J.A., Baugnon K.L., Mullins M.E., Allen J.W., Cao L., Xie Z., Zhang D., Zhao J. (2020). Recent advances in photodynamic therapy based on emerging two-dimensional layered nanomaterials. Nano Res..

[3] Chen C., Wu C., Yu J., Zhu X., Wu Y., Liu J., Zhang Y. (2022). Photodynamic-based combinatorial cancer therapy strategies: Tuning the properties of nanoplatform according to oncotherapy needs. Coord. Chem. Rev..

[4] Benov L. (2015). Photodynamic therapy: current status and future directions. Med. Princ. Pract..

[5] Zhu T.C., Finlay J.C. (2008). The role of photodynamic therapy (PDT) physics. Med. Phys..

[6] Lan Y., Zhu X., Tang M., Wu Y., Zhang J., Liu J., Zhang Y. (2020). Construction of a near-infrared responsive upconversion nanoplatform against hypoxic tumors: Via NO-enhanced photodynamic therapy. Nanoscale.

[7] Castano A.P., Demidova T.N., Hamblin M.R. (2005). Mechanisms in photodynamic therapy: Part three—Photosensitizer pharmacokinetics, biodistribution, tumor localization and modes of tumor destruction. Photodiagnosis Photodyn. Ther..

[8] Nowis D., Makowski M., Stokłosa T., Legat M., Issat T., Gołab J. (2005). Direct tumor damage mechanisms of photodynamic therapy. Acta Biochim. Pol..

[9] Zhu X., Zhang J., Liu J., Zhang Y. (2019). Recent progress of rare-earth doped upconversion nanoparticles: synthesis, optimization, and applications. Adv. Sci..

[10] Sheng T., Xu M., Li Q., Wu Y., Zhang J., Liu J., Zhu X., Zhang Y. (2021). Elucidating the role of energy management in making brighter, and more colorful upconversion nanoparticles. Mater. Today Phys..

[11] Tang M., Zhu X., Zhang Y., Zhang Z., Zhang Z., Mei Q., Zhang J., Wu M., Liu J., Zhang Y. (2019). Near-infrared excited orthogonal emissive upconversion nanoparticles for imaging-guided on-demand therapy. ACS Nano.

[12] Shen Z., Ma Q., Zhou X., Zhang G., Hao G., Sun Y., Cao J. (2021). Strategies to improve photodynamic therapy efficacy by relieving the tumor hypoxia environment. NPG Asia Mater..

[13] Zang L., Zhao H., Ji X., Cao W., Zhang Z., Meng P. (2017). Photophysical properties, singlet oxygen generation efficiency and cytotoxic effects of aloe emodin as a blue light photosensitizer for photodynamic therapy in dermatological treatment. Photochem. Photobiol. Sci..

[14] Song X., Feng L., Liang C., Yang K., Liu Z. (2016). Ultrasound triggered tumor oxygenation with oxygen-shuttle nanoperfluorocarbon to overcome hypoxia-associated resistance in cancer therapies. Nano Lett..

[15] DeClerck K., Elble R.C. (2010). The role of hypoxia and acidosis in promoting metastasis and resistance to chemotherapy. Front. Biosci. (Landmark Ed).

[16] Zannella V.E., Dal Pra A., Muaddi H., McKee T.D., Stapleton S., Sykes J., Glicksman R., Chaib S., Zamiara P., Milosevic M. (2013). Reprogramming metabolism with metformin improves tumor oxygenation and radiotherapy response. Clin. Cancer Res..

[17] Zhang Y., Lei P., Zhu X., Zhang Y. (2021). Full shell coating or cation exchange enhances luminescence. Nat. Commun..

[18] Zhang Y., Zhu X., Zhang J., Wu Y., Liu J., Zhang Y. (2021). Synergistic upconversion photodynamic and photothermal therapy under cold near-infrared excitation. J. Colloid Interface Sci..

[19] Wu C., Wu Y., Zhu X., Zhang J., Liu J., Zhang Y. (2021). Near-infrared-responsive functional nanomaterials: the first domino of combined tumor therapy. Nano Today.

[20] Li Q., Yuan S., Liu F., Zhu X., Liu J. (2021). Lanthanide-doped nanoparticles for near-infrared light activation of photopolymerization: fundamentals, optimization, and applications. Chem. Rec..

[21] Hemmer E., Benayas A., Légaré F., Vetrone F. (2016). Exploiting the biological windows: current perspectives on fluorescent bioprobes emitting above 1000 nm. Nanoscale Horiz..

[22] Blázquez-Castro A. (2019). Optical tweezers: phototoxicity and thermal stress in cells and biomolecules. Micromachines.

[23] Ai X., Mu J., Xing B. (2016). Recent advances of light-mediated theranostics. Theranostics.

[24] Xiang Y., Zheng S., Yuan S., Wang J., Wu Y., Zhu X. (2022). Near-infrared mediated orthogonal bioimaging and intracellular tracking of upconversion nanophotosensitizers. Mikrochim. Acta.

[25] Zhang Z., Jayakumar M.K.G., Zheng X., Shikha S., Zhang Y., Bansal A., Poon D.J.J., Chu P.L., Yeo E.L.L., Chua M.L.K. (2019). Upconversion superballs for programmable photoactivation of therapeutics. Nat. Commun..

[26] Güleryüz B., Ünal U., Gülsoy M. (2021). Near infrared light activated upconversion nanoparticles (UCNP) based photodynamic therapy of prostate cancers: An in vitro study. Photodiagnosis Photodyn. Ther..

[27] Wu J., Du S., Wang Y. (2019). Photosensitizer coated upconversion nanoparticles for triggering reactive oxygen species under 980 nm near-infrared excitation. J. Mater. Chem. B.

[28] Lee S.Y., Lee R., Kim E., Lee S., Park Y.I. (2020). Near-infrared light-triggered photodynamic therapy and apoptosis using upconversion nanoparticles with dual photosensitizers. Front. Bioeng. Biotechnol..

[29] Xie Y., Sun Y., Sun J., Wang Y., Yu S., Zhou B., Xue B., Zheng X., Liu H., Dong B. (2023). Upconversion fluorescence-based PDT nanocomposites with self-oxygenation for malignant tumor therapy. Inorg. Chem. Front..

[30] Dou Q.Q., Teng C.P., Ye E., Loh X.J. (2015). Effective near-infrared photodynamic therapy assisted by upconversion nanoparticles conjugated with photosensitizers. Int. J. Nanomed..

[31] Sabri T., Pawelek P.D., Capobianco J.A. (2018). Dual activity of rose bengal functionalized to albumin-coated lanthanide-doped upconverting nanoparticles: targeting and photodynamic therapy. ACS Appl. Mater. Interfaces.

[32] Han S., Hwang B.W., Jeon E.Y., Jung D., Lee G.H., Keum D.H., Kim K.S., Yun S.H., Cha H.J., Hahn S.K. (2017). Upconversion nanoparticles/hyaluronate–rose bengal conjugate complex for noninvasive photochemical tissue bonding. ACS Nano.

[33] Nahorniak M., Pop-Georgievski O., Velychkivska N., Filipová M., Rydvalová E., Gunár K., Matouš P., Kostiv U., Horák D. (2022). Rose bengal-modified upconverting nanoparticles: synthesis, characterization, and biological evaluation. Life.

[34] Lucky S.S., Muhammad Idris N., Li Z., Huang K., Soo K.C., Zhang Y. (2015). Titania coated upconversion nanoparticles for near-infrared light triggered photodynamic therapy. ACS Nano.

[35] Wang H., Jiang S., Shao W., Zhang X., Chen S., Sun X., Zhang Q., Luo Y., Xie Y. (2018). Optically switchable photocatalysis in ultrathin black phosphorus nanosheets. J. Am. Chem. Soc..

[36] Kim H., Yoon J., Kim H.K., Lee W.T., Nguyen N.T., Le X.T., Lee E.H., Lee E.S., Oh K.T., Choi H.G., Youn Y.S. (2023). Upconverting nanoparticle-containing erythrocyte-sized hemoglobin microgels that generate heat, oxygen and reactive oxygen species for suppressing hypoxic tumors. Bioact. Mater..

[37] Gu T., Cheng L., Gong F., Xu J., Li X., Han G., Liu Z. (2018). Upconversion composite nanoparticles for tumor hypoxia modulation and enhanced near-infrared-triggered photodynamic therapy. ACS Appl. Mater. Interfaces.

[38] Wu X., Yan P., Ren Z., Wang Y., Cai X., Li X., Deng R., Han G. (2019). Ferric hydroxide-modified upconversion nanoparticles for 808 nm NIR-triggered synergetic tumor therapy with hypoxia modulation. ACS Appl. Mater. Interfaces.

[39] Liu Y., Liu Y., Bu W., Cheng C., Zuo C., Xiao Q., Sun Y., Ni D., Zhang C., Liu J., Shi J. (2015). Hypoxia induced by upconversion-based photodynamic therapy: towards highly effective synergistic bioreductive therapy in tumors. Angew Chem. Int. Ed. Engl..

[40] Chen Q., Xie X., Huang B., Liang L., Han S., Yi Z., Wang Y., Li Y., Fan D., Huang L., Liu X. (2017). Confining excitation energy in Er^3+^-sensitized upconversion nanocrystals through Tm^3+^-mediated transient energy trapping. Angew Chem. Int. Ed. Engl..

[41] Wang J., Zheng S., Zhang H., Wu Y., Zhang J., Liu J., Zhu X., Zhang Y. (2023). Unraveling the growth process of core-shell structured upconversion nanoparticles: implications for color tuning of upconversion luminescence. ACS Appl. Nano Mater..

[42] Xu X., Zhou Z., Liu Y., Wen S., Guo Z., Gao L., Wang F. (2019). Optimising passivation shell thickness of single upconversion nanoparticles using a time-resolved spectrometer. APL Photonics.

[43] Brown G.C. (1999). Nitric oxide and mitochondrial respiration. Biochim. Biophys. Acta.

[44] Brunori M., Giuffrè A., Forte E., Mastronicola D., Barone M.C., Sarti P. (2004). Control of cytochrome c oxidase activity by nitric oxide. Biochim. Biophys. Acta.

[45] Sen S., Kawahara B., Chaudhuri G. (2013). Mitochondrial-associated nitric oxide synthase activity inhibits cytochrome c oxidase: Implications for breast Cancer. Free Radic. Biol. Med..

[46] Torresan M.F., Wolosiuk A. (2021). Critical aspects on the chemical stability of NaYF_4_-Based upconverting nanoparticles for biomedical applications. ACS Appl. Bio Mater..

[47] Yu J., Yin W., Peng T., Chang Y.N., Zu Y., Li J., He X., Ma X., Gu Z., Zhao Y. (2017). Biodistribution, excretion, and toxicity of polyethyleneimine modified NaYF_4_:Yb,Er upconversion nanoparticles in mice via different administration routes. Nanoscale.

[48] Zhang Z., Jayakumar M.K.G., Shikha S., Zhang Y., Zheng X., Zhang Y. (2020). Modularly assembled upconversion nanoparticles for orthogonally controlled cell imaging and drug delivery. ACS Appl. Mater. Interfaces.

[49] Yang N., Gong F., Cheng L. (2022). Recent advances in upconversion nanoparticle-based nanocomposites for gas therapy. Chem. Sci..

[50] Liu S., Sun Y., Zhang T., Cao L., Zhong Z., Cheng H., Wang Q., Qiu Z., Zhou W., Wang X. (2022). Upconversion nanoparticles regulated drug & gas dual-effective nanoplatform for the targeting cooperated therapy of thrombus and anticoagulation. Bioact. Mater..

[51] Ai F., Sun T., Xu Z., Wang Z., Kong W., To M.W., Wang F., Zhu G. (2016). An upconversion nanoplatform for simultaneous photodynamic therapy and Pt chemotherapy to combat cisplatin resistance. Dalton Trans..

[52] Zhang P., Steelant W., Kumar M., Scholfield M. (2007). Versatile Photosensitizers for Photodynamic Therapy at Infrared Excitation. J. Am. Chem. Soc..

[53] Zhang X., Tian G., Yin W., Wang L., Zheng X., Yan L., Li J., Su H., Chen C., Gu Z., Zhao Y. (2015). Controllable generation of nitric oxide by near-infrared-sensitized upconversion nanoparticles for tumor therapy. Adv. Funct. Mater..

[54] Vetrone F., Naccache R., Mahalingam V., Morgan C.G., Capobianco J.A. (2009). The Active-Core/Active-Shell Approach: A Strategy to Enhance the Upconversion Luminescence in Lanthanide-Doped Nanoparticles. Adv. Funct. Mater..

