# Supplementary material for: Photoswitchable upconversion nanoparticles with excitation-dependent emission for programmed stepwise NIR phototherapy
Source: iScience. 2023 Sep 9;26(10):107859. doi: 10.1016/j.isci.2023.107859 (PMC10520541; doi:10.1016/j.isci.2023.107859)
Supplement: Document S1. Figures S1–S11 and Table S1 [file mmc1.pdf]

iScience, Volume *26*

Supplemental information

Photoswitchable upconversion nanoparticles with excitation-dependent emission for programmed stepwise NIR phototherapy

Shanshan Zheng, Hengji Zhang, Ting Sheng, Yi Xiang, Jing Wang, Yao Tang, Yihan Wu, Jinliang Liu, Xiaohui Zhu, and Yong Zhang

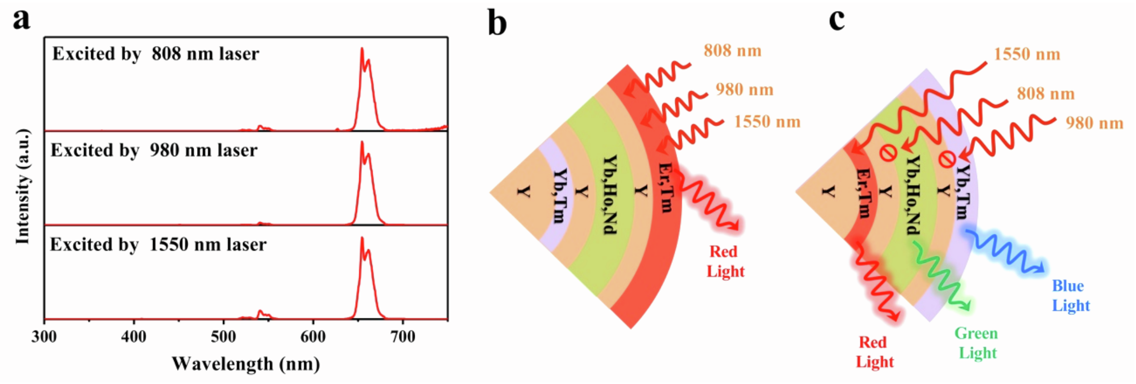

Figure S1. Arrangement of shell structure to achieve orthogonal emissive colors. Related to Figure 1. **(a)**. Upconversion emission spectra of $NaErF_4$:Tm under 808 nm, 980 nm and 1550 nm excitation light. **(b)**. Schematic illustration of the core-multi-shell UCNPs with $NaYF_4$:Yb: Tm in the S1 shell and $NaErF_4$:Tm in the S5 shell. In this design, the $NaErF_4$:Tm shell can be excited by all the three excitation lights and emit red color, therefore affecting the color purity. **(c)**. Schematic illustration of the core-multi-shell UCNPs with $NaErF_4$:Tm in the S1 shell and $NaYF_4$:YbTm in the S5 shell. In this design, the excitation and emission of the three luminescent ions are strictly segmented that enables to release orthogonal trichromatic colors in response to three different NIR lights.

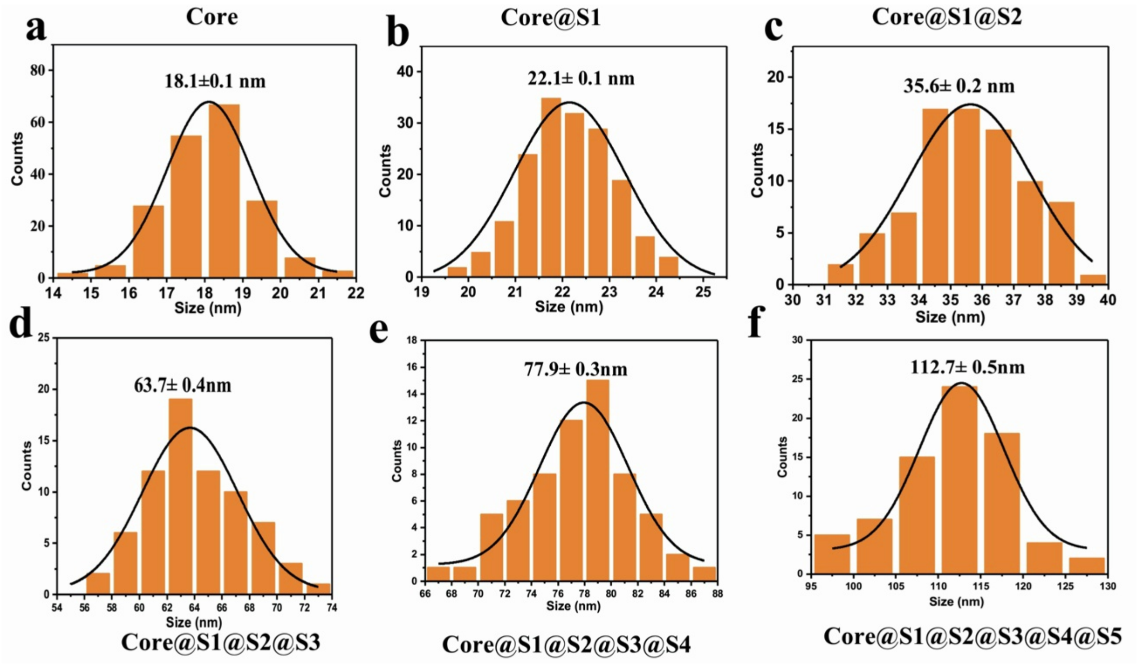

Figure S2. Particle size calculation of UCNP nanoparticles. Related to Figure 2. Particle size distribution of **(a)** Core, **(b)** Core@S1, **(c)** Core@S1@S2, **(d)** Core@S1@S2@S3, **(e)** Core@S1@S2@S3@S4 and **(f)** Core@S1@S2@S3@S4@S5.

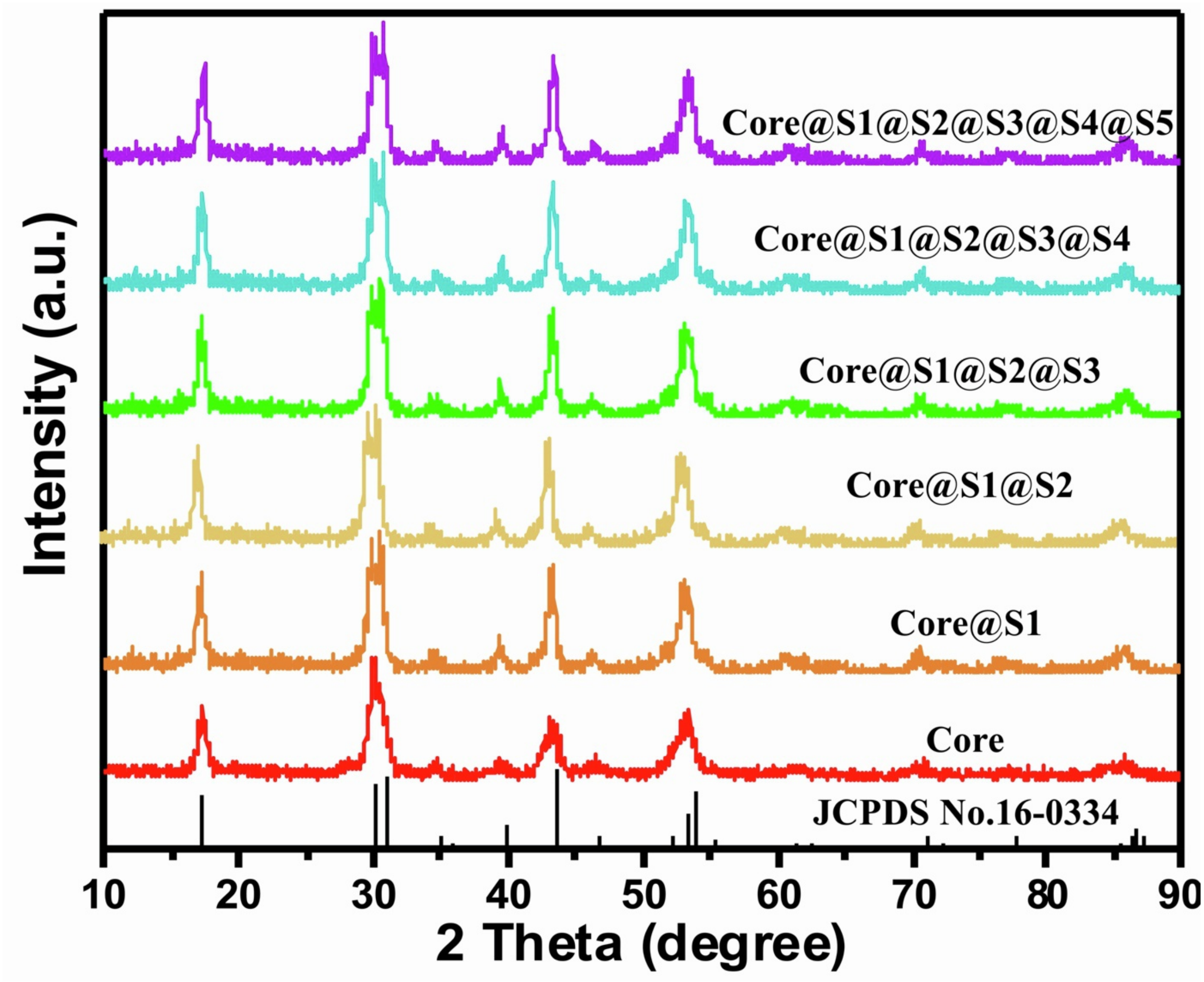

Figure S3. Phase characterization of UCNPs with different shell thickness. Related to Figure 2. XRD patterns of synthesized nanoparticles of Core, Core@S1, Core@S1@S2, Core@S1@S2@S3, Core@S1@S2@S4 and Core@S1@S2@S4@S5.

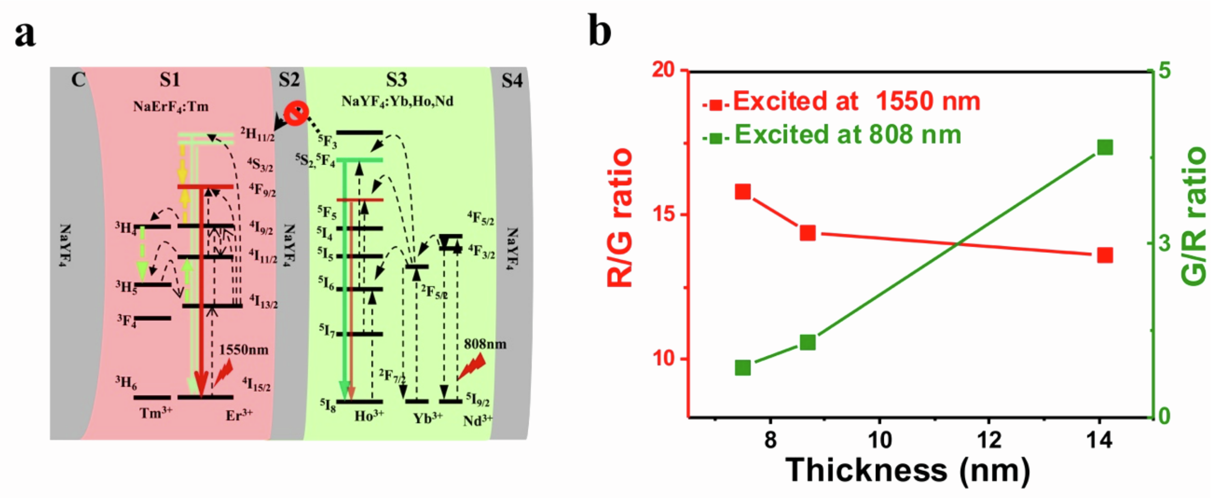

Figure S4. Color tuning of green-emitting portion (C@S1@S2@S3@S4). Related to Figure 3. **(a).** Energy migration mechanism of green-emitting portion (C@S1@S2@S3@S4) under 808 nm light. **(b).** The changes of red to green ratio of UCNPs under illumination by 1550 nm light (red) and green to red intensity ratio under illumination by 808 nm light (green).

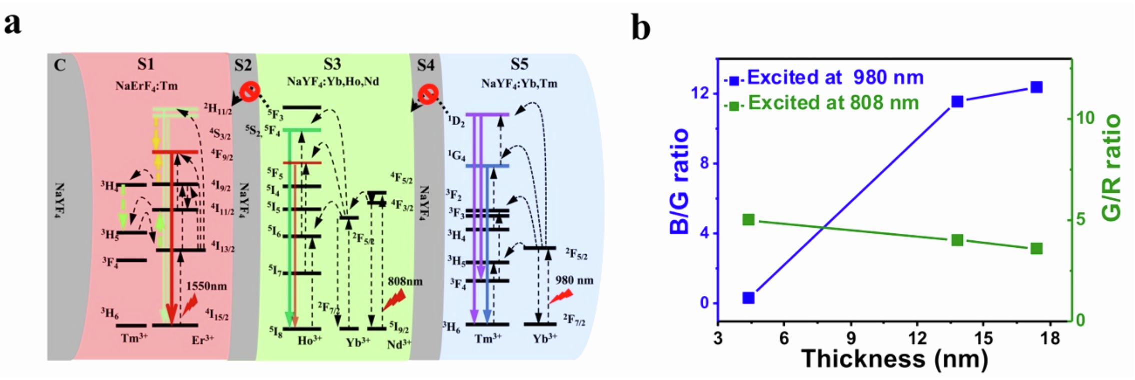

Figure S5. Color tuning of blue-emitting portion (C@S1@S2@S3@S4@S5). Related to Figure 3. **(a).** Energy migration mechanisms of blue-emitting portion (C@S1@S2@S3@S4@S5) under 980 nm light. **(b).** The changes of blue to green ratio (B/G) of UCNPs under illumination by 980 nm light (blue) and green to red (G/R) intensity ratio under illumination by 808 nm light (green).

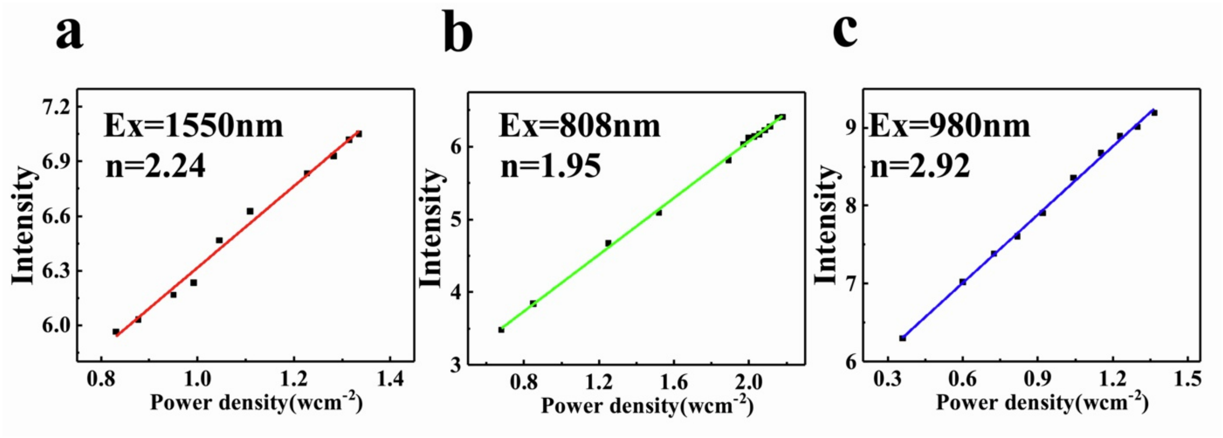

Figure S6. Power dependence of the upconversion emission intensity on excitation light power density with different wavelength. Related to Figure 3. Photon number fitting of UCNPs under 808 nm **(a)**, 980 nm **(b)**, and 1550nm **(c)** excitation.

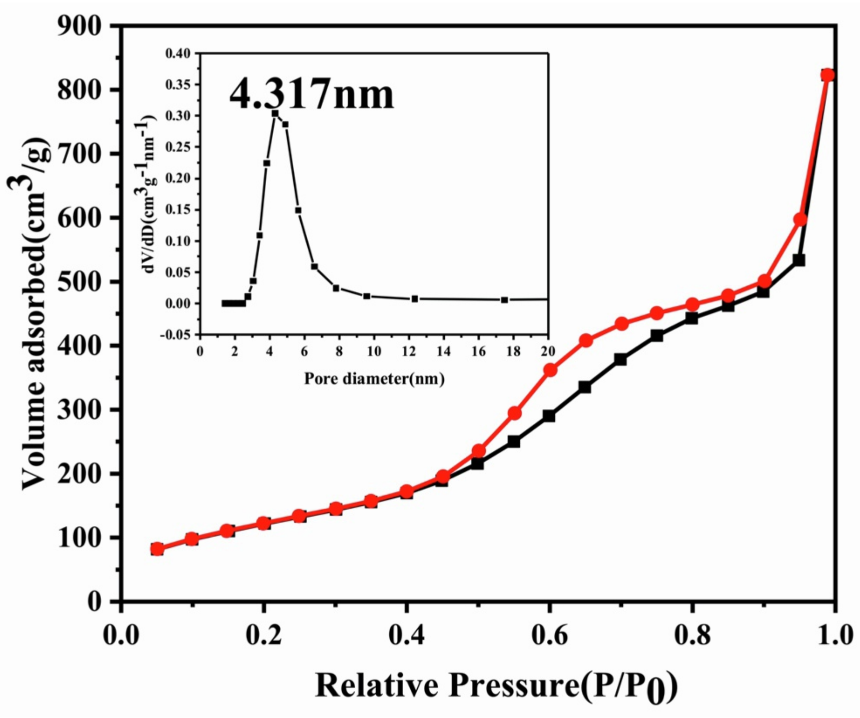

Figure S7. Characterization of mesoporous structure of UCNPs@mSiO$_2$. Related to Figure 4. N$_2$ adsorption/desorption isotherms and pore size distribution curves (inset) of UCNPs@mSiO$_2$.

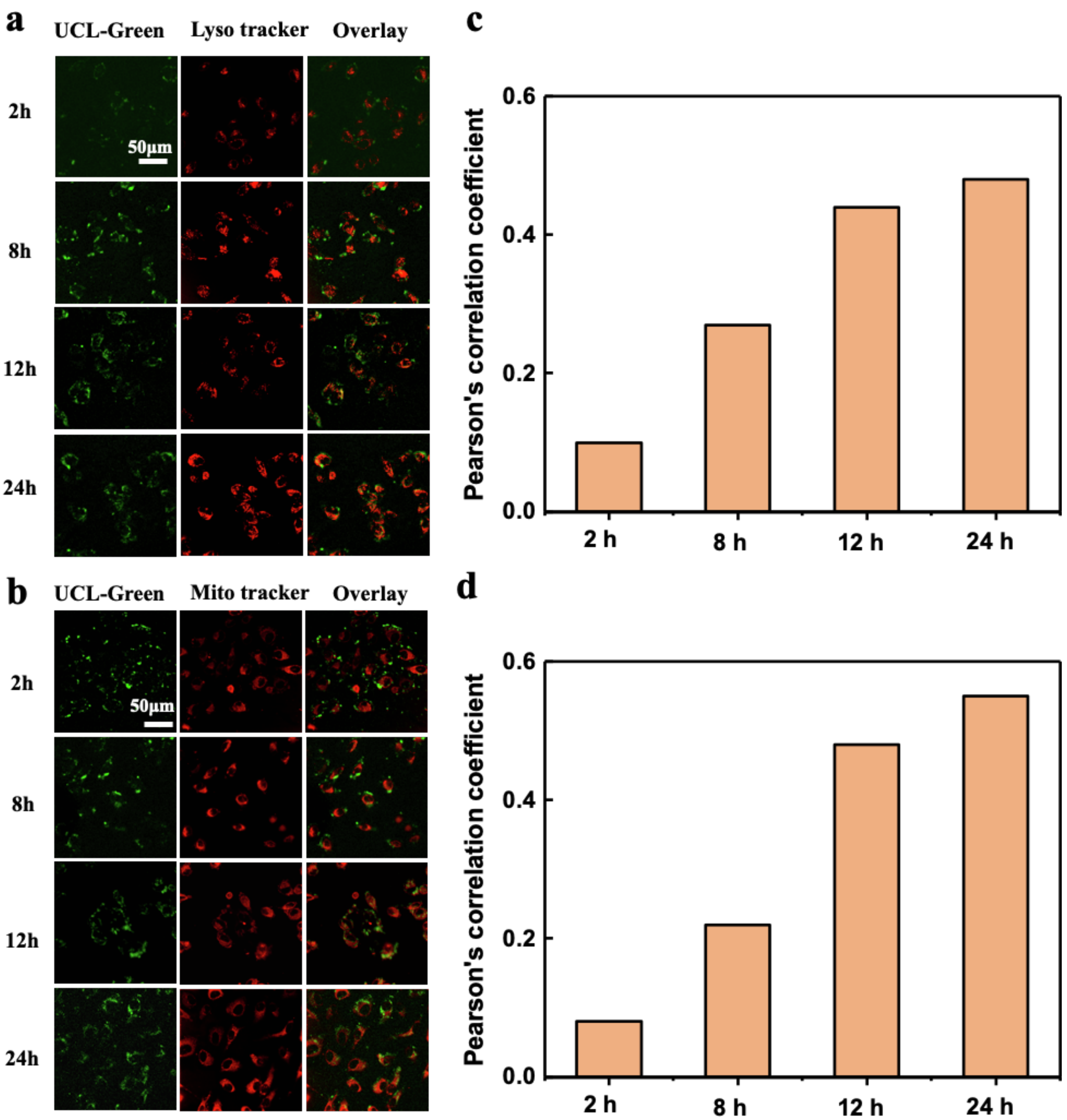

Figure S8. Investigation of intracellular uptake of UCNPs@mSiO$_2$-ZnPc-RBS. Related to Figure 4. Confocal imaging of intracellular uptake of UCNPs@mSiO$_2$-ZnPc-RBS in lysosomes **(a)** and mitochondria **(b)**. The co-localization of UCNPs with lysosome **(c)** and mitochondria **(d)** is analyzed based on Pearson's coefficient method.

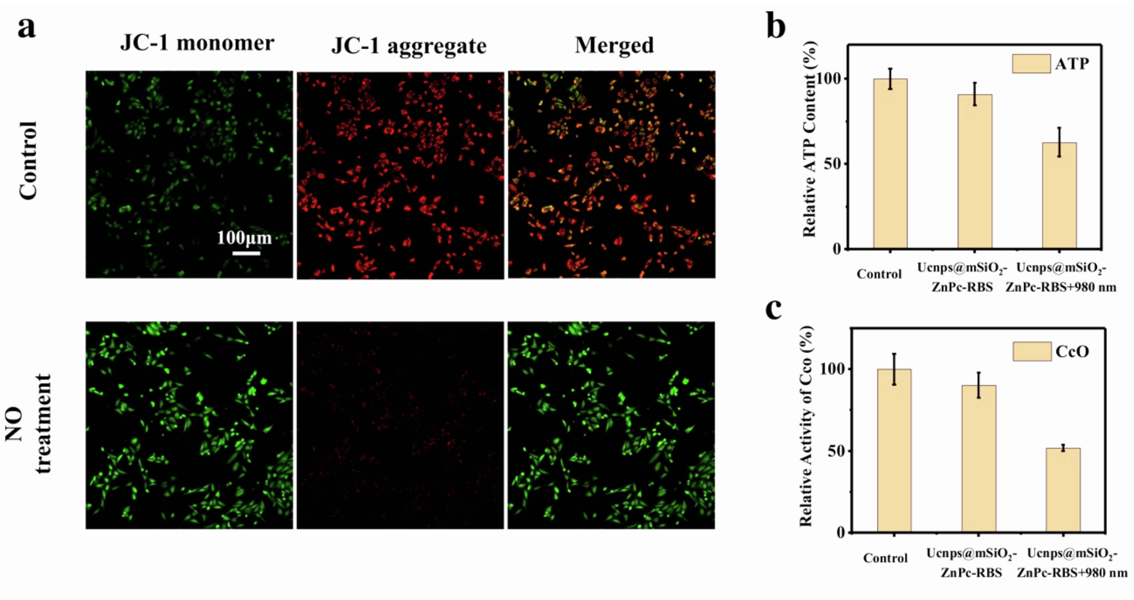

Figure S9. Investigation of NO release on cellular respiration. Related to Figure 5. **(a).** Confocal images of JC-1 fluorescence. Mitochondrial membrane potential of HeLa cells after different treatment was evaluated using the JC-1 indicator. The green fluorescence indicates the monomeric JC-1while red represents JC-1 aggregate. **(b)**. Comparison of relative ATP content in the cells with and without NO treatment. **(c)**. Relative activity of CcO after different treatments.

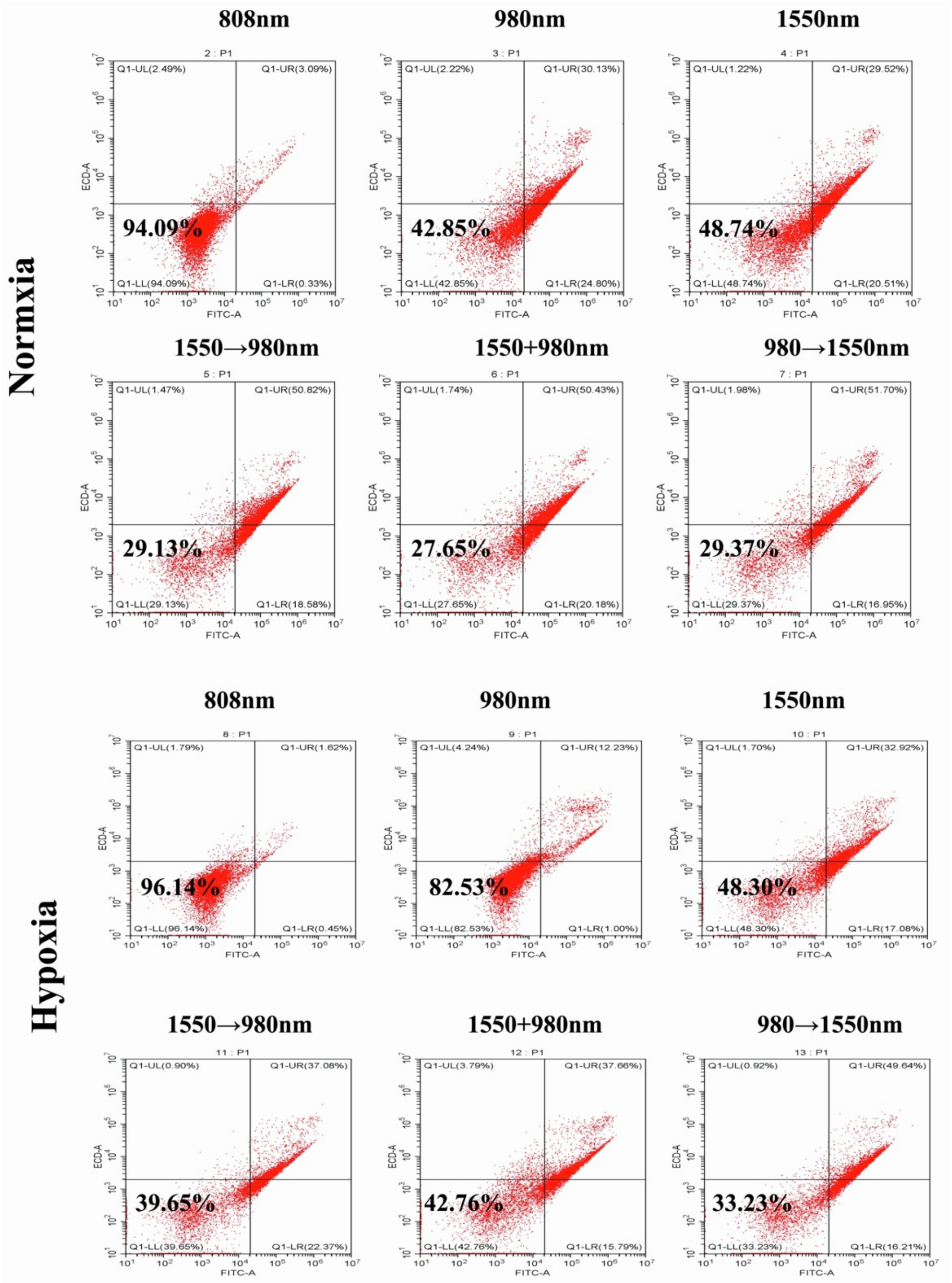

Figure S10. Apoptosis and necrosis study of HeLa cells. Related to Figure 5. Flow cytometry analysis of HeLa cells cultured under different light irradiation in normoxia and hypoxia.

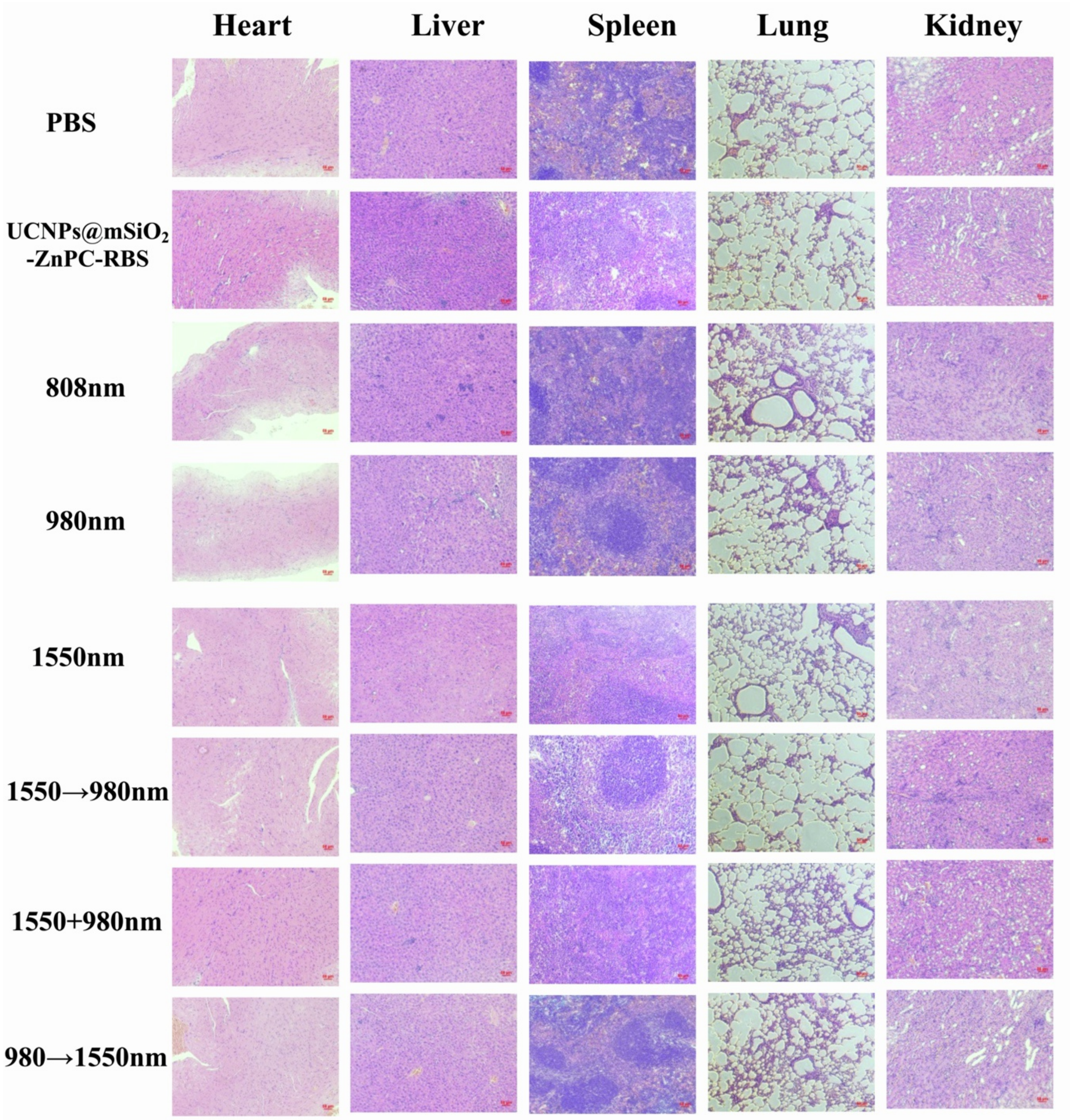

Figure S11. Haematoxylin & Eosin (H&E) staining analysis. Related to Figure 6. Images of major organs (heart, liver, spleen, lung, kidney) of mice stained by H&E in each group.

Supporting Table S1 Summary of NO-based therapy based on UCNP nanocomposites. Related to STAR Methods.

Nanodrug platform	Photosensitive molecules	Stimuli	Released species	Programmable activation (Y/N)	Therapeutic application	Ref.
UCNPs@mSiO$_2$-ZnPc-RBS	RBS/ZnPc	980 nm/808 nm light	NO/ROS	Y	Programmable NO/PDT therapy	This work
UCNPs@mSiO$_2$-CUR-RBS	RBS/CUR	980nm light	NO/ROS	N	NO-enhanced PDT	[S1]
UCNPs@MgSiO$_3$-BNN6/DOX	BNN6	980nm light	NO/DOX	N	NO/chemotherapy	[S2]
T-UCNPs-RBS-DOX	RBS	808 nm light	NO/DOX	N	NO/chemotherapy	[S3]
UCNPs-RBS-DOX	RBS	808 nm light	NO/DOX	N	NO enhanced-chemotherapy	[S4]
USMSs-PEG-SNO	SNO	X-ray	NO	N	NO-sensitized radiotherapy	[S5]
UNTPs-L-αPD-1	mPEG-PNTC-PEI	808 nm light	NO	N	NO-triggered immunotherapy	[S6]
UCPA-BNN	BNN	808 nm light	NO	N	NO therapy	[S7]
UCNPs@M-β-CD-NORMs	[(3)Ru(NO)(Cl)]	980nm light	NO	N	NO therapy	[S8]
UCNP@SiO$_2$@SNAP	SNAP	980nm light	NO	N	NO therapy	[S9]

Abbreviations: UCNPs: upconversion nanoparticles; ZnPc: zinc phthalocyanine; RBS: Roussin's Black Salt([NH_4][$Fe_4S_3(NO)_7$]); CUR: curcumin; BNN6: N,N′-Di-sec-butyl-N,N′-dinitroso-1,4-phenylenediamine; DOX: doxorubicin; T-UCNPs: Tween-20 coated UCNPs; USMSs: UCNPs@SiO$_2$; USMSs-PEG-: PEG-silane modified USMSs; SNO: S-nitrosothiol; UNTPs: up-converted polymeric NO-stabilized nano-gasholder (UNTPs); UNTPs-L: UNTPs upon NIR irradiation; mPEG-PNTC-PEI: polymeric NO donor of poly(ethylene glycol)-poly(nitrate carbonate)-pentaethylenehex-amine; αPD-1: anti-PD-1 antibody; UCPA: UCNP@mSiO$_2$@PAA-Ald; PAA:Poly(acrylic acid); Ald: alendronate sodium trihydrate; M-b-CD: methyl-b-cyclodextrin; NORMs: ruthenium nitrosyl complexes [(3)Ru(NO)(Cl)]; SNAP: S-nitroso-N-acetyl-dl-penicillamine.

Reference:

S1. Lan Y, Zhu X, Tang M, et al (2020) Construction of a near-infrared responsive upconversion nanoplatform against hypoxic tumors via NO-enhanced photodynamic therapy. Nanoscale 12:7875–7887. https://doi.org/10.1039/C9NR10453D

S2. Li S, Song X, Zhu W, et al (2020) Light-Switchable Yolk–Mesoporous Shell UCNPs@$MgSiO_3$ for Nitric Oxide-Evoked Multidrug Resistance Reversal in Cancer Therapy. ACS Appl Mater Interfaces 12:30066–30076. https://doi.org/10.1021/acsami.0c06102

S3. Zhang X, Guo Z, Liu J, et al (2017) Near infrared light triggered nitric oxide releasing platform based on upconversion nanoparticles for synergistic therapy of cancer stem-like cells. Sci Bull 62:985–996. https://doi.org/10.1016/j.scib.2017.06.010.

S4. Zhang X, Tian G, Yin W, et al (2015) Controllable generation of nitric oxide by near-infrared-sensitized upconversion nanoparticles for tumor therapy. Adv Funct Mater 25:3049–3056. https://doi.org/10.1002/adfm.201404402

S5. Fan W, Bu W, Zhang Z, et al (2015) X-ray Radiation-Controlled NO-Release for On-Demand Depth-Independent Hypoxic Radiosensitization. Angew Chemie Int Ed 54:14026–14030. https://doi.org/10.1002/anie.201504536

S6. Li Y, Qian H, Huang X, et al (2022) Up-converted nano-gasholder with precise nitric oxide release remodels immunosuppressive microenvironment and potentiates tumor immunotherapy. Nano Today 42:. https://doi.org/10.1016/j.nantod.2022.101381

S7. Light N, De N, Ye J, et al (2021) Near-Infrared Light and Upconversion Nanoparticle Defined Nitric Oxide-Based Osteoporosis Targeting Therapy. https://doi.org/10.1021/acsnano.1c04974

S8. Zhao J, Hu Y, Lin SW, et al (2020) Enhanced luminescence intensity of near-infrared-sensitized upconversion nanoparticles: Via Ca^{2+}doping for a nitric oxide release platform. J Mater Chem B 8:6481–6489. https://doi.org/10.1039/d0tb00088d

S9. Li C, Shen J, Yang J, et al (2018) NIR-Triggered Release of Nitric Oxide with Upconversion Nanoparticles Inhibits Platelet Aggregation in Blood Samples. 1700281:1–6. https://doi.org/10.1002/ppsc.201700281
